# Organic photodiodes: device engineering and applications

**DOI:** 10.1007/s12200-022-00049-w

**Published:** 2022-12-19

**Authors:** Tong Shan, Xiao Hou, Xiaokuan Yin, Xiaojun Guo

**Affiliations:** School of Electronic Information and Electrical Engineering, Shanghai Jiao Tong University, Shanghai, 200240 China

**Keywords:** Organic photodiodes, Wearable electronics, Photoplethysmography, Optical imagers, Spectrometers, Optical communications

## Abstract

Organic photodiodes (OPDs) have shown great promise for potential applications in optical imaging, sensing, and communication due to their wide-range tunable photoelectrical properties, low-temperature facile processes, and excellent mechanical flexibility. Extensive research work has been carried out on exploring materials, device structures, physical mechanisms, and processing approaches to improve the performance of OPDs to the level of their inorganic counterparts. In addition, various system prototypes have been built based on the exhibited and attractive features of OPDs. It is vital to link the device optimal design and engineering to the system requirements and examine the existing deficiencies of OPDs towards practical applications, so this review starts from discussions on the required key performance metrics for different envisioned applications. Then the fundamentals of the OPD device structures and operation mechanisms are briefly introduced, and the latest development of OPDs for improving the key performance merits is reviewed. Finally, the trials of OPDs for various applications including wearable medical diagnostics, optical imagers, spectrometers, and light communications are reviewed, and both the promises and challenges are revealed.

**Graphical Abstract:**

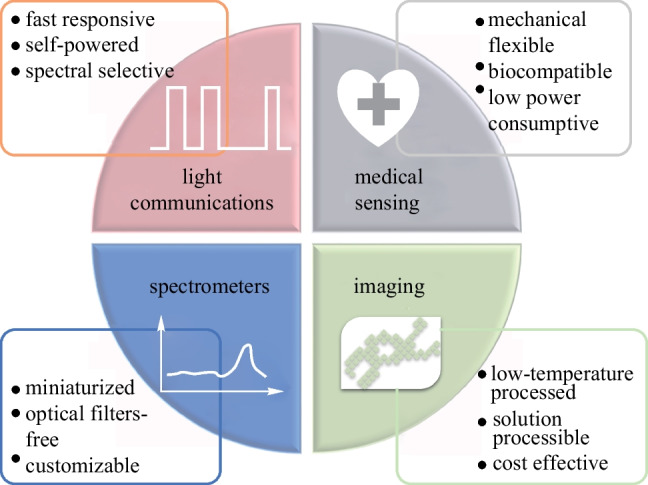

## Introduction

Photodetectors based on various inorganic semiconductors, including silicon, III-V semiconductors, metal oxides, and semiconducting alloys, have been extensively explored in optical sensing or imaging systems for medical, security, and industrial applications [1–5]. With advantages of wide-range tunable photoelectrical properties, low-temperature facile processes, and excellent mechanical flexibility, organic semiconductor (OSC) photodetectors have received wide attention as promising technology choices for developing optical sensing or imaging interfaces in emerging applications where the existing inorganic devices may not meet requirements [1, 6–9]. Among various organic photodetector device configurations, the organic photodiode (OPD) is most widely investigated, due to its fast response, high sensitivity, and making full use of the existing research basis of organic photovoltaics (OPVs) [6, 8, 9]. Extensive research work has been carried out on exploring materials, device structures, physical mechanisms, and processing approaches to improve the performance of OPDs to the level of their inorganic counterparts. To date, OPDs with spectra spanning from ultraviolet (UV) to near-infrared (NIR) have been reported [10–13]. The performance of state-of-art OPDs even rivals that of commercialized low-noise silicon photodiodes (PDs) within the visible spectral range [14]. Large area, ultra-thin and flexible OPDs have also been fabricated, showing benefits for creating optical sensing interfaces in new form factors [15].

Additionally, various system prototypes of OPDs have been built to seek potential applications in wearable health sensing devices, optical imagers, spectrometers, light communication systems, etc. [16–29]. Due to inherent mechanical flexibility and process compatibility with low Young’s modulus plastic substrates, one promising application of OPDs is in skin-conformal optical sensors for wearable health monitoring and medical diagnostics (e.g., photoplethysmography (PPG) and cardiovascular sensing) with minimal invasiveness [15]. With low-temperature facile processes, the OPD is also more suitable than the hydrogenated amorphous silicon (α-Si:H) PD for direct integration on top of thin-film transistor (TFT) backplanes for large-area/flexible high-resolution optical imagers [30]. There has been intensive research on OPD-based active-matrix imagers for medical imaging and biometric authentication applications, which used inorganic TFT backplanes, including α-Si:H, amorphous indium-gallium-zinc-oxide (a-IGZO), and low-temperature polycrystalline silicon (LTPS) to leverage the industry-standard processes [31, 32]. All-organic integration of OPDs on top of the organic TFT (OTFT) backplane was also developed to achieve thermal and mechanical matching of the whole material stack with common plastic films for ubiquitous optical imagers of highly customizable form factors [33, 34]. With tailorable photoelectrical properties, miniaturized spectrometer prototypes were made by integrating customized wavelength-selective OPD pixels into compact modules for handheld or wearable spectrum measurements [35, 36]. Fast response with bandwidth up to MHz and some specific photo-response properties of OPDs make them potential in the development of various light communication systems, including indoor navigation and data communication (high indoor photo-generation efficiency) [37], encryption communication (spectral selectivity) [38, 39], multi-channel visible light communication (multi-wavelength response) [40], and remote control (NIR response) [41].

The research progress as shown above has established a solid material and device basis for developing high-performance OPDs for integrated systems, and has also presented great promise of OPDs for many emerging applications. There have been several reviews in the literature on the advances of OPD-related research, covering topics of materials, device structures, physics, processing methods, and applications with OPDs, respectively [1, 6, 7, 9, 42–45]. It is vital to link the device optimal design and engineering to the system requirements, and examine the existing deficiencies of OPDs for practical applications. Therefore, this review will start from discussions on the required performance metrics for different applications. Then the fundamentals of the OPD device structures and physics are briefly introduced, and the latest development of OPDs for improving the key performance metrics is reviewed. Finally, the trials of OPDs for various applications are reviewed, and both the promises and challenges are revealed.

## Performance metrics

Defining proper performance metrics is key to link device-level optimal design to specific application requirements. The key metrics for typical optical sensing or imaging applications should cover performance in four aspects: photon-to-electron conversion efficiency, transient response, detection range, and spectral responsivity. High photon-to-electron conversion efficiency is generally required for all kinds of applications. Fast transient response is important when details of time-domain optical signals are required to be sampled or high-speed information transmission is needed, with typical applications including optical communication, PPG, and dynamic image/video capturing. To realize high-quality imaging, the capability of detecting optical signals across a wide intensity range is also pivotal. High spectral responsivity is necessary for color recognition or imaging, spectrum analysis, and optical signal processing. In the following, the key metrics will be discussed.

### Responsivity (*R*)

The ratio of the output photocurrent (*I*_ph_, or photo-induced current in specific PDs) to the input optical power (*P*_in_), defined as responsivity (*R*), is used to represent the photon-to-electron conversion efficiency. *R* is wavelength dependent, and can be calculated from the measured external quantum efficiency (EQE) as follows:

1 $$R={I}_{\mathrm{ph}}/ {P}_{\mathrm{in}}=\mathrm{EQE}\cdot \lambda q/ (hc),$$

where *q* is the elementary electric charge, *λ* is the wavelength of the incident photon, *h* is the Planck constant, *c* is the speed of light. EQE is described as the ratio of the number of charge carriers collected to the number of incident photons.

### Noise equivalent power (NEP) and specific detectivity (D*)

The noise equivalent power (NEP), is the incident optical power, which generates a photocurrent equal to the noise of the photodetector. This value is used as the minimum detectable incident power.

The NEP is the common metric that quantifies a photodetector’s sensitivity by measuring the weakest optical signal that can be detected. The NEP can be defined as the input optical power that results in a signal-to-noise ratio (SNR) of 1 for 1 Hz bandwidth, and is expressed as:

2 $$\mathrm{NEP}={P}_{\mathrm{in}}/(\mathrm{SNR}\cdot \sqrt{\mathrm{BW}})={i}_{\mathrm{in},\mathrm{N}}/(R\cdot \sqrt{\mathrm{BW}}),$$

where ${i}_{\mathrm{in},\mathrm{ N}}$ is the noise current, and BW is the normalized bandwidth.

Therefore, NEP is given in watt per square root of hertz (W/$\sqrt{\rm Hz}$). It is desirable to have a NEP as low as possible since a low value corresponds to a lower noise floor and therefore a more sensitive detector. Even at higher input intensities, a low NEP is beneficial for lower noise in the output signal.

As described in Eq. (2), NEP is largely limited by the noise current. Even when no optical input is applied to a photodetector, there will be some amount of generated current noise that results in a certain average output noise power. Typically, the noise current ${i}_{\mathrm{in},\mathrm{ N}}$ is composed of three parts: flicker noise (1/*f* noise), Johnson-Nyquist thermal noise, and dark shot noise [46, 47]. The 1/*f* noise is frequency-dependent and is most dominant at low frequencies. The thermal and shot noises are frequency independent and scale with the shunt resistance and dark current density of the device, respectively.

To more intuitively evaluate the capability of detecting weak optical signals, the specific detectivity (D*) is defined as the reciprocal of NEP normalized by the square root of the device active area (*A*):

3 $${D}^{*}=\sqrt{A}/ \mathrm{NEP}= R\sqrt{A\cdot \mathrm{BW }}/ {i}_{\mathrm{in},\mathrm{N}},$$

where D* allows performance comparison of different PDs, regardless of the operating mechanism and active area, and a higher D* indicates a better detection performance of the photodetector.

### Linear dynamic range

The range of the input light intensity that can be detected is another important metric for a photodetector and can be derived as the ratio of the maximum detectable photocurrent to the noise current when there is no input light signal. For practical applications, a linear relationship between the photo-current and the incident light power is convenient for calibration and signal processing. However, the linear relationship is only valid for a limited range of the input signal intensity. At high intensity of the input signal, physical limitations of the device may cause saturation of the output signal, where the photocurrent increases nonlinearly with the input optical intensity and gradually approaches a constant value. The linear dynamic range (LDR) is thus defined as:

4 $$\mathrm{LDR}=20\mathrm{log} ({L}_{\mathrm{max}}/ {L}_{\mathrm{min}}),$$

where *L*_min_ and *L*_max_ are the lowest and the highest incident optical intensity in the linear range of photocurrent.

To a certain extent, subsequent processing with non-linear function fitting can compensate for the saturation effects and thus extend the dynamic range. High LDR is important for high-performance imaging, as the minimum quantitatively measurable light intensity can be much smaller than the maximum one, leading to imaging with a large grayscale range.

### Response speed and bandwidth

Fo﻿r dynamic imaging and high-frequency optical signal detection, rapid response is important for the photodetector. The response time (rise or fall time) and the − 3 dB bandwidth (*f*_*−*3 dB_) are common metrics in the time domain and frequency domain, respectively. The rise (or fall) time is defined as the time duration when the normalized photocurrent rises from 10 to 90% (or decays from 90 to 10%). The rise (or fall) time within the order of tens of μs is sufficient for PPG and typical dynamic imaging applications.

Response bandwidth, *f*_*−*3 dB_ is recognized as the frequency at which the photocurrent drops to − 3 dB (half) of the low frequency value, which is highly related to the carrier transit time (*τ*_tr_) and the circuit resistance–capacitance (RC) time constant as given by

5 $${{f}_{-3\mathrm{ dB}}}^{-2}={\left(3.5/ (2\uppi {\tau }_{\mathrm{tr}})\right)}^{-2} + {\left(1/(2\mathrm{\pi RC})\right)}^{-2}.$$

According to the NIR communication protocol, *f*_*−*3 dB_ over 38–50 kHz is the precondition for the application of PDs to NIR light communications [41]. Higher response bandwidth over hundreds of MHz is desirable for high-speed light communications [21].

### Spectrum responsivity

**Fig. 1:**
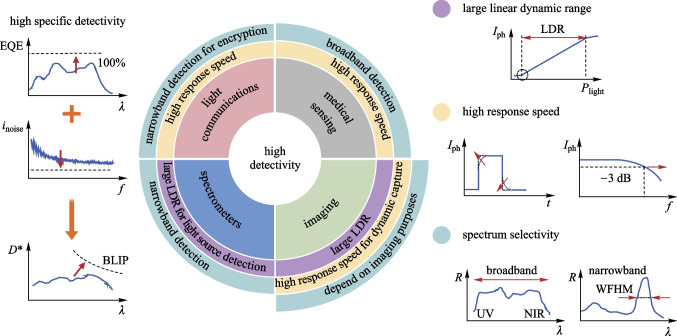
Illustration of required performance metrics of OPDs for different applications

One of the advantages of OSCs is the tunability of the absorption spectrum. Either broadband OPDs, covering the range from UV to NIR, or narrowband devices can be made by choosing appropriate OSCs and device structures. As shown in Fig. 1, the full width at half maximum (FWHM) of the primary detection peak is used to evaluate the selectivity of PDs. A narrower FWHM denotes better selectivity. To quantitatively evaluate the spectra selectivity, the spectral rejection ratio (SRR) is introduced as:

6 $$\mathrm{SRR}={R}_{\mathrm{peak}}/ {R}_{\mathrm{out}},$$

where *R*_peak_ is the peak responsivity in the target spectral window and *R*_out_ is the responsivity outside the window. Small FWHM and large SRR at different wavelengths are critical for PDs to be used for spectrometer applications. NIR responsivity is required for blood oxygen measurements, bioimaging, spectrometers, and NIR light communications.

In addition to the above figures of merit, there are other derived performance parameters in the practical devices, which are discussed below.

## Device fundamentals

### Device structure and material stack

**Fig. 2:**
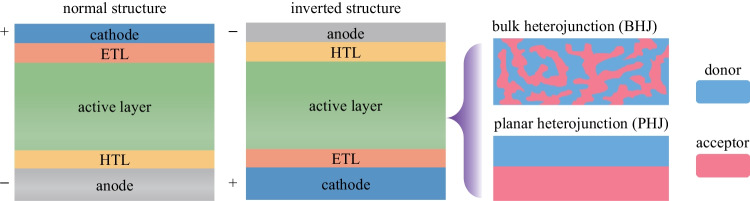
Schematic structure of the normal and inverted structure OPDs with the planar heterojunction and the bulk heterojunction for the active layer

The typical OPD architectures comprise a photo-active layer, sandwiched between an anode and a cathode, with a hole transport layer (HTL) and an electron transport layer (ETL) for efficient charge extraction, as shown in Fig. 2. Depending on the relative location of the anode and the cathode, OPDs can be fabricated in either a normal structure with a bottom anode and top cathode or an inverted one with a top anode and bottom cathode. Since the exciton binding energy of OSCs is relatively large (> 0.3 eV) due to their low dielectric constant, building a p-n heterojunction similar to that in inorganic semiconductor devices is effective for separating photo-generated electron–hole pairs into free charges. Either a planar heterojunction (PHJ) or a bulk heterojunction (BHJ) composed of donor and acceptor OSCs can be used to form the active layer (Fig. 2). The BHJ possesses an interpenetrating network of donors and acceptors. Compared with the PHJ, it can provide more sufficient donor/acceptor interfacing to harvest more excitons and generate a larger number of photo-generated electron–hole pairs [48, 49]. Therefore, the BHJ structures have been widely adopted for OPVs and OPDs and can be formed through various solution coating approaches using blended solutions of donor and acceptor OSCs [50, 51].

The material stacks used for OPDs have been derived largely from the extensive OPV research [17, 52]. For the BHJ active layer, poly(3-hexylthiophene) (P3HT), a thiophene-based polymer, remains one of the best donor materials for its good hole mobility and facile synthesis and processing, and fullerene derivatives, such as [6, 6]‐phenyl‐C61 butyric acid methyl ester (PC_61_BM) and indene-C60 bis-adduct (ICBA), are predominantly used as acceptors for their decent electron mobility [53, 54]. A thicker active layer is beneficial to reduce leakage current and improve light absorption, but it might affect charge extraction due to the limited carrier mobility. Therefore, the high mobility donor OSCs developed for OPVs with improved packing order and stronger intermolecular interactions have been explored for OPDs [52]. The non-fullerene acceptors developed for OPVs have also been shown to realize a high molar extinction coefficient and high crystallinity [55–57]. The optical and electrical characteristics of OSCs can be finely tuned by modulating their conjugation length and alternating electron-push and electron-pull building blocks to enhance intramolecular charge transfer [58, 59]. The resultant hybridization of molecular orbitals endows them with intense absorption bands and high oscillator strengths enabling substantial absorption in the fabricated thin-film devices. Hence, broadband OPDs with good performance metrics in the UV to NIR range have been realized [10, 11].

Besides the active layer, the interfacial layers including the HTL and ETL are also critical to the overall OPD performance. Ideal interfacial layers should have high conductivity and have energy levels matching with the active layer and the contact electrodes for efficient charge transport and extraction. Process compatibility with both the upper and lower layers within the material stack is also vital. To avoid influence on light absorption in the active layer, the interfacial layer on the light transmission path needs to be highly transparent to light of a certain wavelength. The interfacial layer materials include small molecule or polymer OSCs and inorganic metal oxides or metal salts [60]. For examples, poly(3,4-ethylenedioxythiophene) doped with poly (styrene sulfonate) (PEDOT:PSS), nickel oxide (NiO_*x*_), molybdenum oxide (MoO_*x*_) are common HTL materials [61–63], and polyethyleneimine ethoxylated (PEIE), poly((9,9-bis(30-(N,N-dimethylamino)propyl)-2,7-fluorene)-alt-2,7-(9,9-dioctylfluorene)) (PFN), zinc oxide (ZnO), titanium oxide (TiO_2_), and stannic oxide (SnO_2_) are commonly used for ETLs [64–69].

Many reported OPDs are of the bottom-illuminated structure, typically employing indium tin oxide (ITO) as the transparent bottom electrode. However, to develop high-resolution active-matrix imagers, top-illuminated OPD pixels need to be fabricated on top of the TFT backplane to obtain a large aperture ratio [30, 31]. For that, various transparent electrode technologies, including ultrathin metals, conductive metal oxides, conductive polymer, and metal nanowire networks, can be adopted [70–72]. Among them, ultra-thin metal (e.g., Ag) or alloy deposited by thermal evaporation is a promising choice due to relatively simple and mature processes for scalable manufacturing [73, 74]. An additional thin oxide capping layer (e.g., MoO_*x*_, WO_3_) is typically used to reduce the reflection by the ultra-thin metal and to enhance light absorption [75–78].

### Operation mechanism

**Fig. 3:**
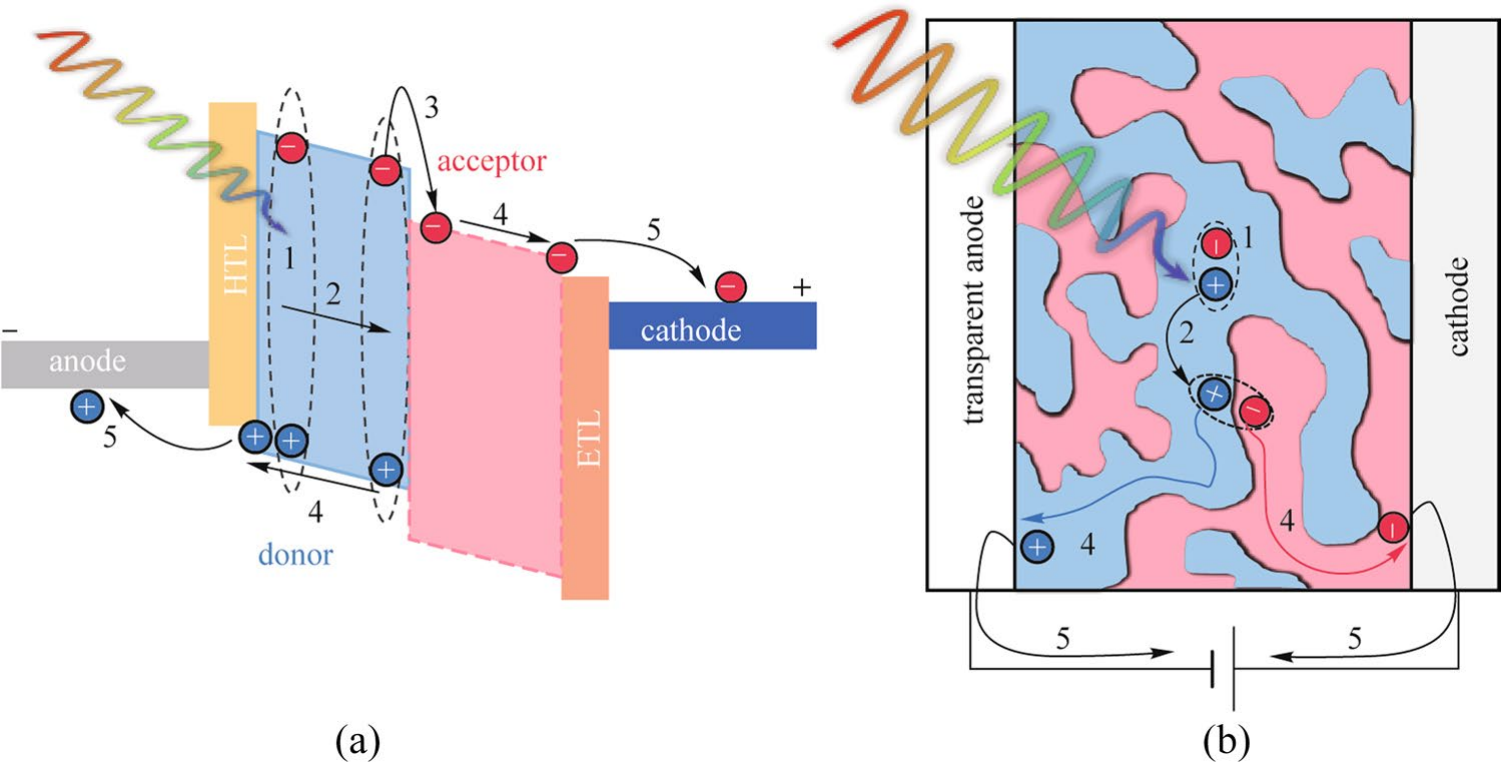
**a** Energy diagrams and **b** working mechanisms for a general type OPD

The basic operating mechanism of an OPD is illustrated in Fig. 3, involving five steps: (1) exciton generation in the active layer upon light illumination; (2) exciton diffusion to the donor/acceptor interface; (3) exciton dissociation to free charges (electrons and holes); (4) charge carrier transport through the HTL and ETL; and (5) charge collection by the contact electrodes to form the photon-generated current.

To achieve high EQE and thus high responsivity, photoactive OSCs with high oscillator strengths are needed to ensure efficient exciton generation. Then the morphology of the BHJ structure (i.e. the donor and acceptor domain sizes) needs to be well controlled to enhance exciton diffusion to the donor/acceptor interface before recombination. Moreover, energy level matching between donors and acceptors, and the formation of bi-continuous high-mobility donor/acceptor networks is important to strengthen exciton dissociation and promote charge carrier transport. Further, proper interfacial layers are required to form low contact resistance between the active layer and the electrodes for efficient charge collection, and also to block the transport of the opposite type carriers for low dark current. Efficient BHJ systems with proper material stack design enable OPDs to achieve a maximum EQE exceeding 80% even at an external bias of zero volt (photovoltaic mode) [79, 80]. Typically, the OPDs are reverse-biased, which can facilitate the separation of hole/electron pairs and charge transport underan external electric field [71]. However, the increase of dark current with the applied reverse bias voltage due to the charge carrier injection needs to be suppressed [46]. In a word, the photoactive layer is the most fundamental part to determine the key performance metrics of OPDs, while the overall structure and the interfacial layers are vital for maximizing the performance.

## Device engineering for performance improvement

The EQE and the responsivity of the OPDs are mainly determined by the photo-active layers. There has been plenty of research on OSCs for the photo-active layers in OPVs, which can be also adopted for developing OPDs with large photon-to-electron conversion efficiency [81–84]. In this part, device engineering approaches for improving the OPD performance are reviewed.

### Reduction of dark current and noise

When efficient photoactive layers are adopted to achieve high EQE and large responsivity, both the detectivity and LDR are limited by the dark shot noise current floor [18]. Various strategies have thus been developed to reduce the dark current of OPDs, including minimizing the trap density and energetic disorder in the OSC layers [85], optimization of the phase morphology in the active layer [86], and interfacial engineering to suppress external charge injection [60]. Further, a thick active layer is also needed to reduce the leakage current for large-area integration, but this might reduce the responsivity and the response speed [8, 87]. Huang et al. reported an OPD with an ultra-narrow bandgap non-fullerene acceptor CO1-4Cl for NIR (920–960 nm) detection [88]. ITO/ZnO bottom cathode, blended active layer of PTB7-Th:CO1-4Cl and MoO_*x*_/Ag top anode were applied in an inverted structure. With such a device structure, it is demonstrated that a thicker active layer obviously reduced the dark current, but with a certain sacrifice of the responsivity and the EQE. The resulting D* is as high as 10^12^ Jones in the visible to the NIR spectrum range, which is close to that of a commercial silicon PD.

**Fig. 4:**
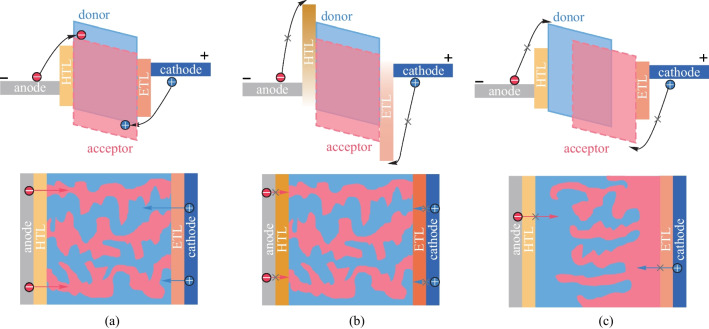
Schematics show the charge injection process in OPDs, and two universal strategies for preventing charge injection: **a** common BHJ with percolating networks, **b** BHJ with blocking layers, and **c** quasi-planar heterojunction with vertical phase segregation

In an OPD with a well-blended BHJ, the intrinsic dark current under reverse bias might come from the undesired charge carrier injection from the contact electrodes, including movement of electrons from the anode to the lowest unoccupied molecular orbital (LUMO) of the acceptor, and holes from the cathode into the highest occupied molecular orbital (HOMO) of the donor. Figure 4(a) shows schematically the charge injection process in OPDs. To eliminate the undesired charge injection paths for reducing the dark current, one way is to enhance the blocking of undesired charge injection at the contacts (Fig. 4(b)), the other is to form a quasi-planar heterojunction (q-PHJ) active layer (Fig. 4(c)).

1. Improving charge selectivity at the contacts

The first strategy to reduce dark current is improving charge selectivity at the contacts [52]. As shown in Fig. 4b, raising the energy barriers of charge injection by selecting a charge transport layer with reasonable energy levels can suppress unwanted charge injection while facilitating the extraction of photo-generated carriers.

**Fig. 5:**
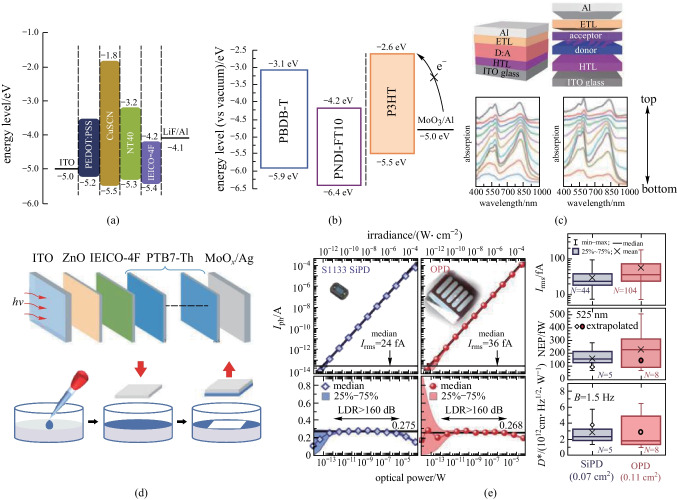
Representations on reduction of dark current and noise. **a** CuSCN with high LUMO energy was applied as the EBL instead of the generally used PEDOT:PSS to reduce dark current by two orders of magnitude. Reproduced with permission from Ref. [96]. **b** Inserting P3HT between the active layer and the anode as an EBL decreased the dark current by one order of magnitude. Reproduced with permission from Ref. [97]. **c** Using the sequential deposition method to obtain a q-PHJ active layer. The vertical phase segregation structure effectively reduced the dark current by one order of magnitude. Reproduced with permission from Ref. [94]. **d** Using the water-transfer printing technique to obtain a q-PHJ active layer, and the total noise density was successfully suppressed. Reproduced with permission from Ref. [95]. **e** State-of-the-art OPD enabled competitive device performance compared to silicon PDs. Reproduced with permission from Ref. [14]

Taking the HTL as an example, the LUMO energy should be high enough to elevate the injection energy barriers, while the HOMO energy should maintain an energy cascade between the active layer and the corresponding electrodes so that photo-generated hole collection is not impeded [53]. In other words, the HTL also serves as an electron blocking layer (EBL). Therefore, it is challenging to find suitable interfacial layers with appropriate energy levels and carrier mobility [54–57]. Despite of the variety of appropriate electron transport layers (ETLs) that can block hole injection simultaneously, more and more efforts have been made to develop a solution-processable HTL with sufficient electron blocking ability [58]. Recently, CuSCN was used as the EBL in a NIR-OPD reported by Huang et al. [96]. As shown in Fig. 5a, a higher electron-injection barrier between the anode and the active layer was formed due to the higher conduction band energy level of CuSCN compared to that of the more widely used PEDOT:PSS. The dark current was in turn dramatically reduced by two orders of magnitude. Meanwhile, CuSCN increased the depletion width, leading to an improved D*. Other than the exploration of new interfacial layers, certain organic photosensitive semiconductors were also tried as multifunctional blocking layers. As shown in Fig. 5b, Xu et al. reported flexible all-polymer OPDs incorporating the common donor material P3HT between the active layer and the anode as an EBL, which could decrease the dark current by one order of magnitude [97].

2) Quasi-planar heterojunction (q-PHJ) active layer

The other approach to eliminate the undesired charge injection paths is to create a q-PHJ with control of phase segregation as shown in Fig. 4(b). In the structure, pure donor/accepter phases were formed in the regions near the anode/cathode, respectively, to effectively suppress undesired charge injection, while the center region having mixed donor/acceptor interfaces for substantial photocurrent generation. To obtain such a q-PHJ, sequential deposition of the small molecule acceptor and the polymer donor and morphology engineering were developed [89–93]. Wei et al. recently fabricated a self-powered NIR OPD using the sequential deposition method [94]. As shown in Fig. 5c, due to the formed vertical phase segregation structure, the dark current was effectively reduced by one order of magnitude compared to that of the conventional structures. Additionally, such a q-PHJ could be more stable in morphology, leading to better device stability. To deposit the small molecule acceptor before the polymer donor, Xiong et al. used a water transfer printing technique to avoid dissolving the underneath acceptor layer [95]. The fabricated NIR-OPDs, consisting of a small molecule acceptor (IEICO-4F) layer and multiple polymer donor (PTB7-Th) layers (Fig. 5d), achieved extremely low noise current with suppressed dark current, improved D* and LDR, and faster response simultaneously.

Recently, it has been shown that, with a proper material stack and elaborate processes, the fabricated OPDs can achieve a similar level of noise current, and in turn NEP and D*, to that achieved with the state-of-the-art silicon PD (Fig. 5e; Table 1) [14].

**Table 1:** Performance metrics and experimental approaches of OPDs reported in representative works on dark current (*J*_d_) reduction

Features	Active layer	*J*_d_ (bias)^a^ /(A⋅cm^−2^)	D* (*λ*_max_)^b^ /Jones	LDR /dB	Refs.
Thick BHJ active layer	PTB7-Th:CO1-4Cl	7.0 × 10^–6^ (− 2 V)	3.3 × 10^13^ (940 nm)	126	[88]
PEIE as HBL	PBDTTT-C:PC_71_BM	2.0 × 10^–9^ (− 2 V)	8.5 × 10^12^ (680 nm)	140	[98]
PEIE as HBL	P3HT:ICBA	3.6 × 10^–13^ (0 V)	3.0 × 10^12^ (870 nm)	160	[14]
MEH-PPV as EBL	Squaraine:PC_61_BM	2.0 × 10^–9^ (− 1 V)	3.4 × 10^12^ (700 nm)	NA	[99]
Poly-TPD as EBL	PDPP3T:PC_71_BM	6.3 × 10^–9^ (− 0.5 V)	1.5 × 10^13^ (820 nm)	148	[100]
TIPS-P as EBL	PIDT-TPD:PC_61_BM	1.1 × 10^–9^ (− 5 V)	1.4 × 10^13^ (610 nm)	NA	[101]
CuSCN as EBL	NT40:IEICO-4F	2.7 × 10^–10^ (− 0.1 V)	4.4 × 10^13^ (870 nm)	123	[96]
P3HT as EBL	PBDB-T:PNDI-FT10	1.1 × 10^–8^ (− 3 V)	5.8 × 10^12^ (650 nm)	105	[97]
PTB7-Th as EBL	PTB7-Th:IEICO-4F	1.1 × 10^–6^ (− 0.5 V)	8.8 × 10^11^ (860 nm)	152	[95]
q-PHJ active layer	PTB7-Th:IEICO-4F	2.1 × 10^–11^ (0 V)	2.0 × 10^14^ (805 nm)	83	[94]

^a^Dark current density at a certain bias

^b^Maximum specific detectivity at corresponding wavelength

### Narrowband detection

OPDs with narrowband detection capability have been intensively studied for many applications, such as color imaging [102, 103], spectral measurement [35, 36, 104], and encrypted light communication [40, 105]. Firstly, the molecule structures of the OSCs could be tailored so that the photosensitive active layers can achieve narrowband absorption in a wide spectrum from UV to NIR [106, 107]. However, it is challenging to find donor and acceptor pairs that match in both absorption wavelength and energy levels for the required performance. Moreover, with the absorption redshift to NIR, narrowband absorption with small enough FWHM is more difficult to achieve [38, 108].

**Fig. 6:**
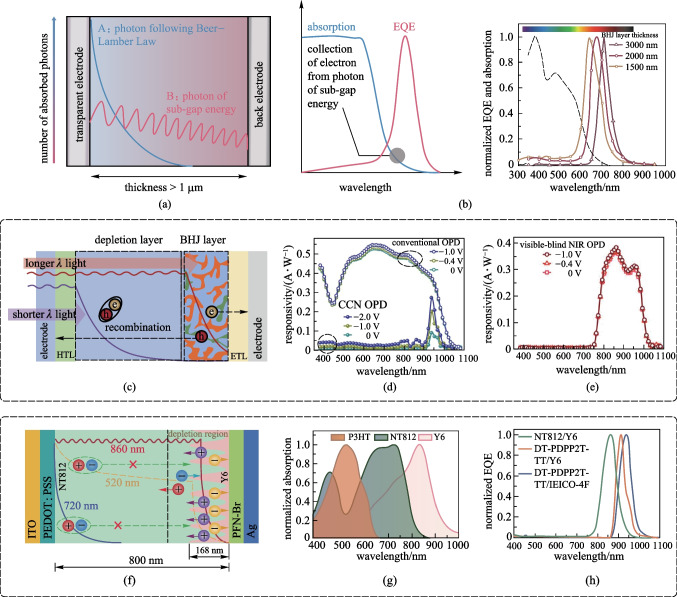
Working principles of narrowband detection OPD by charge collection narrowing (CNN). **a** Working principle of narrowband response for CNN: the absorbed photon distribution for selected wavelengths (A denotes short-wavelength light, and B denotes sub-gap energy light). **b** Increasing the thickness of the active layer to obtain bathochromic-shifted EQE spectra with narrower FWHM. Reproduced with permission from Ref. [109]. **c** Applying the same donor material in the BHJ layer as the light depletion layer to realize narrowband-detective OPD by SF-CCN strategy. **d** Too thick active layer in CCN-type OPDs reduced the responsivity compared to that of conventional OPDs. **e** SF-CCN strategy reduced the visible light crosstalk without sacrificing *R* overmuch. **c**–**e** Reproduced with permission from Ref. [105]. **f** Building a q-PHJ active layer with the light depletion layer of the donor by sequential processing method to realize narrowband-detective OPD by SF-CCN strategy. **g** Applying self-filtering HTL (P3HT) with complementary absorption could further reduce the visible light crosstalk. **h** Band selectivity with narrow FWHM of EQE were achieved based on other donor/acceptor combinations, demonstrating the good universality of this approach. **f**–**h** reproduced with permission from Ref. [111]

To overcome the material limitation, device structure engineering approaches by utilizing charge collection narrowing (CCN) and optical cavity effects were developed [108]. The fundamental mechanism of CCN is to manipulate efficient charge collection locations in the active layer [109]. As shown in Fig. 6a, in a sufficiently thick active layer (i.e., > 1 µm), the incoming photons with a shorter wavelength are fully absorbed in the region near the transparent front electrode (case A). In turn, the photo-generated carriers will recombine before reaching the back reflecting electrode. On the other hand, photons of sub-gap energy (case B) can penetrate the entire active layer and reach the back reflecting electrode, so that the generated carriers can be efficiently extracted. As a result, a peak located near the absorption edge of the active layer appears in the photo-response spectra. As shown in Fig. 6b, by varying the thickness of the active layers, the extraction of charge generated by specific photons can be slightly tuned, resulting in a bathochromic-shifted peak with thicker active layers. Similar to the concept of CCN, charge injection narrowing (CIN) was introduced by Wang et al., which combines the idea of CCN with photomultiplication to achieve narrowband response and charge tunneling-enhanced EQE simultaneously [110].

An issue with the conventional CCN strategy is the necessity of a thick active layer (i.e., > 1 µm), which reduces the responsivity and response speed. This might be circumvented by inserting a shorter-wavelength semiconducting light depletion layer at the front end. Excitons generated by high-energy photons in the light depletion layer cannot be separated into free charges because of the large binding energy and small diffusion length of the photogenerated Frenkel excitons. Only low-energy photons can penetrate the donor/acceptor interface region and generate free charges for collection. The resulted thinner active layer structure is beneficial to promoting the photo-response speed compared to the CCN method. Based on this concept, Lan et al. fabricated a filter-free visible-blind NIR OPD, as shown in Fig. 6c [105]. The same donor material in the BHJ layer was used to build the light depletion layer, and the rear BHJ layer was transferred and printed onto the light depletion layer. Such a structure is called self-filtering CCN (SF-CCN). The light depletion layer not only fully absorbs the shorter-wavelength light, but also served as an HTL to assist charge carrier collection for a higher *R* (Fig. 6d, e). Xie et al. proposed a similar method to manipulate the dissociation region of Frenkel excitons intentionally, as shown in Fig. 6f [111]. The device was fabricated based on a hierarchical structure composed of thick wide-bandgap donor layers followed by narrow bandgap acceptor layers, which were sequentially deposited. This approach is simpler than the former one in directly forming a well-penetrative donor/acceptor BHJ. EQE over 60% at 860 nm with an FWHM of around 50 nm was achieved. To further suppress the EQE response within the spectra range of 500–600 nm, P3HT with complementary absorption to NT812:Y6 film was selected to replace PEDOT:PSS as the self-filtering HTL (Fig. 6g). P3HT was photo-crosslinked to prevent it from being washed away by the subsequent use of solution. The strong electron blocking capability of P3HT contributed to a lower dark current, leading to a D* over 10^13^ Jones. NIR-OPDs based on other donor/acceptor combinations also showed band selectivity with narrow FWHM and D* over 10^13^ Jones, demonstrating the good universality of this approach (Fig. 6h). Such a self-filtering strategy has also been used for narrowband detection OPDs by other groups [112, 113].

**Fig. 7:**
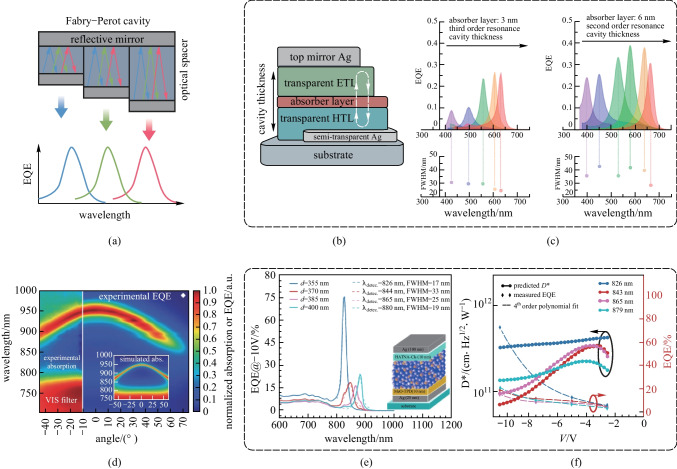
Working principle and representative works of narrowband OPD by an optical resonant cavity. **a** Working principle of Fabry–Perot cavity: the length of optical spacer determines the resonance wavelength. **b** Building a Fabry–Perot cavity in an OPD, where the bottom mirror (electrode) is partially transparent. Changing the cavity thickness could tune the wavelength-selective response. **c** Tunable monochromatic light OPDs within a full visible light detection window was realized by altering the thickness of ETL and HTL. The active layer is 3 nm thick in third-order resonance case, and 6 nm thick in second-order resonance case. The FWHM values can be further reduced from second to third order resonance. **b**, **c** Reproduced with permission from Ref. [119]. **d** Spectrum of device absorption and EQE were angular dependent in cavity-enhanced devices. Reproduced with permission from Ref. [120]. **e** Combining the optical resonant cavity with the photomultiplication effect to boost the spectra selectivity and responsivity. Excellent NIR selectivity implies charge transfer states can trigger photomultiplication as well. **f** Device performance of cavity-enhanced photomultiplication OPD could be optimized by altering the active layer thickness and operating voltage. **e**, **f** Reproduced with permission from Ref. [121]

Another issue of the CCN method is that the spectral tunability is relatively limited, and good SRR can only be achieved at a small voltage bias since the charge separation generated by undesired photons is unavoidable under a large bias. However, the responsivity of the CCN-type OPDs is relatively low at a small voltage bias, making them unsatisfactory for practical applications. Therefore, a more universal technique to enhance spectra selectivity is considered to be design of an optical resonant cavity (ORC), or microcavity, for optical tuning [114–116]. As illustrated in Fig. 7a, the mechanism is based on the incorporation of a Fabry–Perot (FP) cavity formed by two mirroring metal electrodes of which one is semi-transparent. The cavity path length (thickness of the spacer) and refraction index of the spacer stack sandwiched between the two electrodes determine the resonance wavelength, at which the optical transmission is strongly enhanced [117, 118]. For instance, Wang et al. fabricated cavity-enhanced OPDs with a photo-absorbing layer thickness on the nanometer scale, as shown in Fig. 7b [119]. Tunable monochromatic light OPDs within a full visible light detection window was realized by ultrathin organic active layer with thick transparent charge transport layers in a cavity architecture. Undesired resonance overtone signals were avoided by optimizing the location of the active layer within the cavity. FWHM values were between 28 and 43 nm in the second-order resonance and further decreased to 25 nm in the third-order resonance, which is on a par with optical-filtered broadband PDs (Fig. 7c). Although the active layers were only a few nanometers in thickness, the thick transport layer ensured that the OPDs achieved a shunt resistance of 1 MΩ/cm^2^, leading to a D* over 10^12^ Jones.

Even though the optical cavity approach can provide monochromatic detection at a broader range of wavelength, it is still challenging to realize high D* and good spectrum selectivity simultaneously. It should be noted that the detection spectrum is dependent on the angle of light incidence in cavity-enhanced devices, as depicted in Fig. 7d, which makes the cavity design circumscribed for applications where light incidence is omnidirectional [120]. Kublitski et al. recently reported narrowband NIR-OPDs with microcavity enhanced multiplication [121]. As shown in Fig. 7e, with a device structure of Ag (25 nm)/MeO-TPD/ZnPc:C_60_ (3 wt%)/HATNA-Cl_6_/Ag (100 nm), the resonant wavelength was tuned by modulating the active layer thickness from 355 to 400 nm. The EQE enhancement was extended to the charge transfer absorption region and the device presented ultra-narrow FWHM of 17 nm at 826 nm, indicating that charge transfer states also triggered photomultiplication. The combination of optical microcavities and photomultiplication effect boosted the performance of OPDs with NIR selectivity. The optimization of active layer thickness and operation voltage enabled a high D* of 6 × 10^11^ Jones (Fig. 7f; Table 2).

**Table 2:** Performance metrics and working mechanism of OPDs reported in representative works on narrowband detection

Mechanism	Active layer materials	Target peak (FWHM)^a^ /nm	EQE /%	D* (bias)^b^ /Jones	Refs
Narrowband absorption	1(Pyrl):1(Hex):C60	754 (11)	14	1.1 × 10^10^ (0 V)	[107]
CCN	PCDTBT:PC71BM	670 (85)	35	1.8 × 10^12^ (− 1 V)	[109]
CIN	P3HT:PC_71_BM	650 (28)	5.4 × 10^4^	1.4 × 10^11^ (− 60 V)	[110]
CIN	TAPC: C60	335 (27)	5.2 × 10^5^	1.5 × 10^14^ (− 30 V)	[11]
SF-CCN	P3HT:PTB7: PC_71_BM	745 (50)	4.5	1.1 × 10^12^ (0 V)	[112]
SF-CCN	PBDB-T:m-ITIC	700 (120)	53	8.3 × 10^11^ (0 V)	[105]
SF-CCN	NT812:Y6	860 (72)	61	1.2 × 10^13^ (− 0.1 V)	[111]
SF-CCN	DT-PDPP2T-TT:Y6	920 (43)	NA	7.4 × 10^12^ (− 0.1 V)	[111]
SF-CCN	DT-PDPP2T-TT:IEICO-4F	955 (66)	NA	1.6 × 10^13^ (− 0.1 V)	[111]
SF-CIN	P3HT:PC_71_BM	650 (29)	600	7.8 × 10^10^ (− 20 V)	[113]
ORC	PBTTT:PC61BM	775 (15)	40	3.6 × 10^12^ (0 V)	[36]
ORC	PBTTT:PC61BM	960 (17)	24	1.0 × 10^13^ (0 V)	[36]
ORC	DCV5T-Me:C_60_	425 (31)	9	> 10^12^ (0 V)	[119]
ORC	DCV5T-Me:C_60_	630 (25)	26	> 10^12^ (0 V)	[119]
ORC	ZnPc:C_60_	877 (37)	10	1.0 × 10^11^ (0 V)	[122]
CIN and ORC	ZnPc:C_60_	826 (17)	~ 10	6.0 × 10^11^ (− 3.5 V)	[121]

^a^Detection target peak and corresponding full width at half maximum

^b^Maximum specific detectivity at corresponding bias

### Improving response speed

**Fig. 8:**
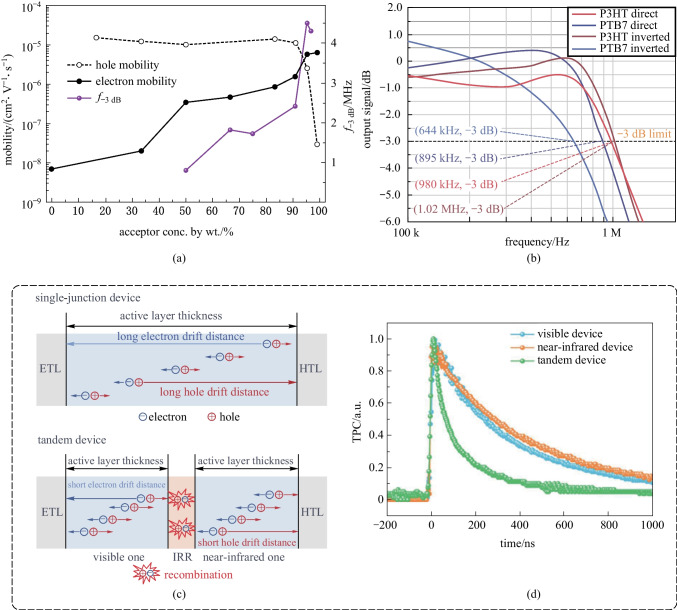
Representative relating to improvement of response speed. **a** Balancing the electron/hole mobilities to boost the response bandwidth of OPDs by altering the blend-ratios of donor and acceptor. Reproduced with permission from Ref. [124]. **b** Response bandwidth of P3HT and PTB7-Th based OPDs with inverted device structures were higher than that of normal device structures. Reproduced with permission from Ref. [125]. **c** Applying tandem device structure to shorten the carriers’ drift distance in each cell, reducing both the *τ*_tr_ and RC time constant. **d** Response time (decay time of the transient photocurrent) of the tandem device was much shorter than that of a single-junction device. **c**, **d** Reproduced with permission from Ref. [126]

Fast response of OPDs is necessary for light communications and real-time imaging or video recording. Due to the relatively low charge carrier mobility of OSCs compared to that of inorganic semiconductors, the achievable response speed of OPDs lags behind that of inorganic PDs. Various attempts have thus been devoted to improving the mobility of the OSCs for faster response OPDs [124]. Generally, the photocurrent decay time after interrupting the irradiated light, which is affected by the charge extraction efficiency, determines the photo-response speed. Suganuma et al. proposed a method introducing multilayered HTLs with a stepwise energy profile to effectively reduce the decay time without affecting the sensitivity of OPDs [123]. Strobel et al. developed fast-response OPDs with response bandwidth up to 3.5 MHz under a bias voltage of − 2 V, due to the use of the high hole mobility donor polymer PIF and the favorable morphology of the active layer [40]. The relationship between D* and operating frequencies was discussed in that work, which is not a major issue except in high-frequency applications. The dynamic performance was limited by the noise current in the lower frequency regime and by the response bandwidth at higher frequencies. Saggar et al. suggested that the balance of the electron/hole mobilities is critical to boosting the response time of OPDs [124]. As shown in Fig. 8a, the cross-over point of the electron/hole mobilities occurred at an acceptor concentration of ~ 95 wt%, and the highest response bandwidth of 4.5 MHz was then obtained.

The response time is also related to the device structure. Salamandra et al. claimed that the inverted device structure was favorable to achieve a larger response bandwidth [125]. As shown in Fig. 8b, no matter whether with P3HT or PTB7, the inverted device exhibited a faster response. In addition, the intrinsic limit of boosting the response speed is the tradeoff between the carrier transit time *τ*_tr_ and the RC time constant. Reducing the thickness of the active layer can reduce the *τ*_tr_, but might cause an increase in the capacitance and dark current [127, 128]. To overcome the dilemma, Liu et al. reported a fast-response OPD with a tandem structure, which reduced both the *τ*_tr_ and RC time constant simultaneously [126]. Figure 8c shows the tandem OPD consisting of a visible absorption front junction and a NIR absorption rear junction with a wide detection range from 300 to 1000 nm. Compared with the single junction OPD with a thicker active layer, the tandem structure reduced the free carrier drift distance and achieved a shorter response time of 146.8 ns (Fig. 8d). With the multi-level barrier enhancement and voltage division, the noise current in the tandem structure OPD was reduced as well.

### Photomultiplication OPDs

**Fig. 9:**
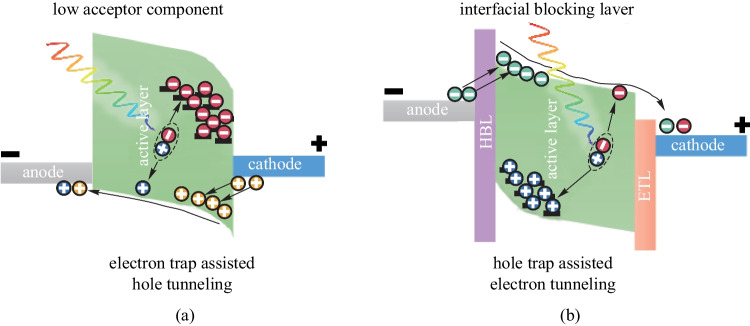
Energy diagrams and working mechanisms for photomultiplication OPD: **a** utilizing ultra-low concentration of one component (with the case of acceptor as an example here) and **b** utilizing interfacial blocking layer (with the case of the hole blocking layer as an example here)

The conventional OPD has a limited EQE less than unity. As a consequence, when an OPD of a small photosensitive area (e.g., a pixel in a high-resolution imager) is used to detect weak optical signals, the generated photocurrent is too small to be measured [35, 36]. To enhance the weak light detection capability, photomultiplication is desired to provide internal photocurrent gain [37]. However, unlike the inorganic counterparts, avalanche photomultiplication is difficult to be achieved due to the relatively large exciton binding energy of OSCs [38]. A basic idea to achieve photomultiplication in OPDs for a photocurrent gain is creating trap-assisted tunneling of the opposite type of charge carriers in the structure. For that, various methods can be used to form charge trapping sites at the interface between the active layer and the electrode, and two common ones are depicted in Fig. 9. One method is to form isolated islands in the BHJ using a donor or accepter of ultra-low concentra^129^tion, so that the dissociated charge carriers are difficult to move to the collecting electrode due to the discontinued transport pathways []. As shown in Fig. 9a, with acceptor to form the charge trapping sites at the active layer/cathode interface, the accumulation of electrons introduced localized electric field, and in turn an interfacial energy level bending. Hole tunneling was then able to occur due to the interfacial energy level bending and the external reverse bias. The other approach relies on the interposition of interfacial blocking layer. As an instance, Fig. 9b shows hole buildup at the interface between the active layer and the HBL, which typically has a very deep HOMO energy level to prevent hole transportation. Eventually, electron tunneling injection can occur. Both inorganic nanoparticles and quantum dots have been employed to provide additional carrier traps in organic BHJ, which also enables carrier tunneling at the Schottky junction [130]. Since the total current in photomultiplication OPDs is created by the photo-generated carriers as well as those injected via tunneling, a photocurrent gain for an EQE higher than unity can be achieved [39]. Nevertheless, these photomultiplication OPDs typically suffer from relatively large dark current and long response time, thus hindering their practical applications [131, 132].

## System integration and applications

Based on the attractive features of OPDs, there have been extensive research efforts on exploring OPDs for various potential system applications including wearable medical diagnostics, optical imagers, spectrometers, and light communications.

### Wearable medical diagnostics

**Fig. 10:**
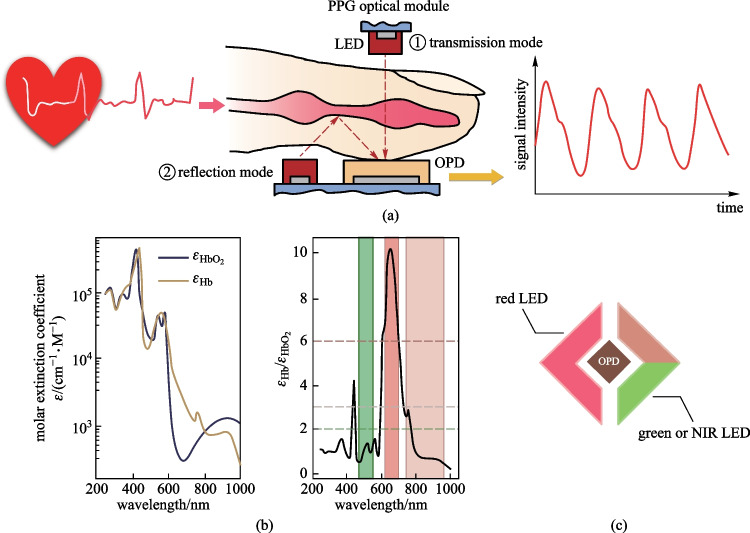
Monitoring heart rate and blood oxygen saturation by PPG. **a** Working principle of PPG: transmission and reflection mode. **b** Molar extinction coefficients of Hb and HbO_2_ at different wavelengths. The distinct ratio of the molar extinction coefficients of Hb and HbO_2_ at red and green or the red and NIR can be used to estimate blood oxygen saturation. Reproduced with permission from Ref. [135]. **c** Schematic diagram of an oximetry system containing an OPD and two LEDs

There is an increasing demand for smart and unobtrusive medical monitoring devices that can accurately detect physiological signals continuously without affecting daily activity. The PPG is a commonly used optical approach for that purpose, and works by measuring the amount of light absorption by the blood volume (Fig. 10a). The functional hemoglobin transport oxygen in the blood circulation system and the oxygen saturation (SO_2_) in tissues can be estimated by optically quantifying the concentration of oxyhemoglobin (HbO_2_) and deoxy-hemoglobin (Hb) [133]. As shown in Fig. 10b, the molar extinction coefficients of HbO_2_ and Hb are different distinctly over the green and red or the red and NIR regions, and can be used for the oximetry. Considering the contrast in molar extinction coefficients and penetration ability to human tissues, the combination of red and NIR is more competent (Fig. 10c). Currently, commercial PPG systems normally consist of inorganic PDs with filters and light-emitting diodes (LEDs) with specific peak emission wavelengths for different measurement purposes. PPG basically have two implementation modes depending on the relative locations of PD and LED, i.e., the transmission mode and reflection mode. The transmission mode requires the tissues to be partially transparent, and the reflection mode is thus more popular [134].

**Fig. 11:**
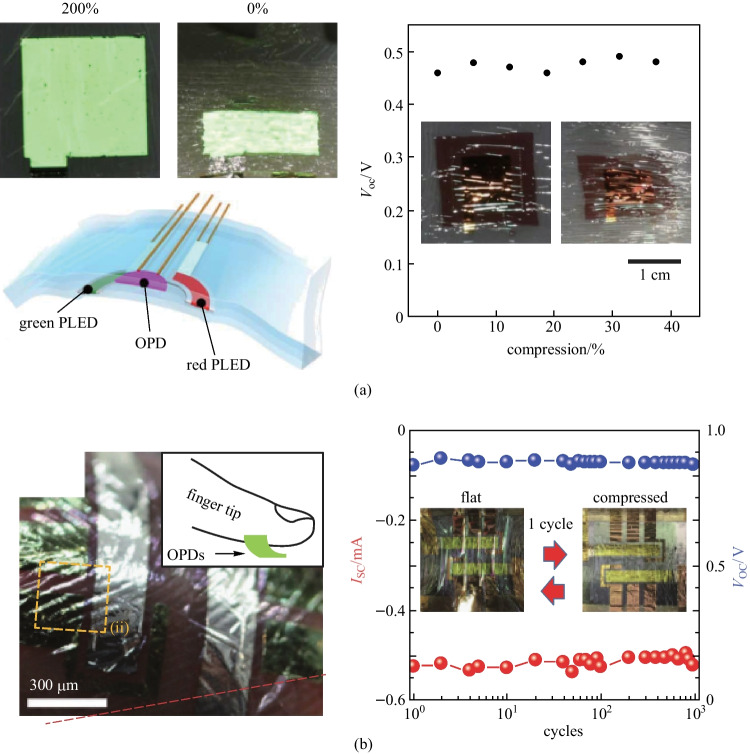
Flexible OPD-based PPG systems. **a** Ultra-flexible reflective pulse oximeter based on an OPD and two polymer LEDs adhered to a prestretched elastomer in flat and wrinkled state. The integrated system could maintain normal operation in both flat and wrinkled state. Reproduced with permission from Ref. [138]. **b** Ultra-thin, flexible, and fingerprint-conformal NIR-OPD for PPG measurement: the OPD could maintain stable operation in 10^3^ cycles when stretching at 100% tensile strain. Reproduced with permission from Ref. [15]

To obtain more conformable on-skin PPG systems for wearable applications, OPDs have been fabricated on an ultra-thin flexible substrate and integrated with light sources of either LEDs or organic LEDs (OLEDs), showing their capability of detecting theheart rate, blood oxygen content, and blood pressure for medical diagnostics [135–137]. Yokota et al. fabricated an ultra-flexible reflective pulse oximeter based on an OPD with a structure of ITO/P3HT:PCBM/MoO_*x*_/Au on a PI planarized perylene substrate [138]. The device presented stable performance when the bending radius dropped down to 100 μm. As shown in Fig. 11a, an integrated system composed of OPD and polymer LEDs on an elastic substrate could maintain normal operation at a tensile strain of 200%. Park et al. reported an ultra-thin (< 3 μm) flexible OPD with a structure of ITO/ZnO/active layer/MoO_*x*_/Ag on a SU-8 planarized perylene substrate for PPG detection (Fig. 11b) [15]. The use of narrow bandgap PIPCP for the active layer enabled an absorption peak near 800 nm. The flexible device presented good operational stability in 1000 bending cycles at a bending radius of 3 μm.

**Fig. 12:**
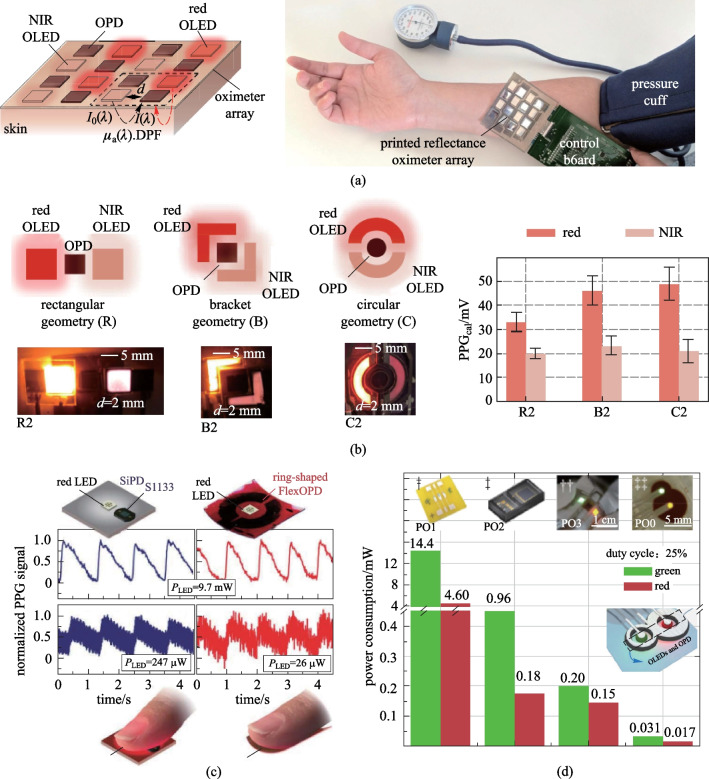
Progress in OPD-based reflectance PPG systems. **a** Flexible reflectance oximeter system integrating multiple OPDs, red OLEDs, and NIR OLEDs for monitoring the change in oxygen saturation. Such OPD and OLED arrays can provide 3 × 3 oximeter pixels. Reproduced with permission from Ref. [135]. **b** Detection efficiency of the PPG system can be optimized by changing sensor geometries. The circular geometry enables larger improvement of the detected signals compared to rectangular and bracket ones. Reproduced with permission from Ref. [139]. **c** OPD with low noise and low NEP can reduce the power consumption in PPG measurement. The minimum required output power of red LED in the ring-shaped flexible OPD-based PPG system dropped to roughly one tenth compared with the case using a commercial silicon PD. Reproduced with permission from Ref. [14]. **d** Geometry of OPD wrapped around each small circular OLEDs is used to achieve the lowest power consumption in PPG measurement. Reproduced with permission from Ref. [140]

OPDs also provide benefits for achieving large area customizable integration. Khan et al. reported a flexible reflectance oximeter system integrating 8 OPDs, 4 red OLEDs, and 4 NIR OLEDs, as shown in Fig. 12a [135]. When used for measuring oxygen saturation on the forehead, the oximeter presented a high accuracy of detection with 1.1% mean error. It is expected to improve the detection accuracy by measuring signals at different locations with an OPD array. Moreover, it was found that the geometries of OPDs and OLED light sources could be optimized to improve the detection performance [139]. Compared with the rectangular geometry, the bracket and circular designs enabled improvement of detected signals by 39.7% /18.2% and 48.6%/ 9.2% in the red/NIR channels, respectively (Fig. 12b).

For wearable continuous monitoring, reducing the power consumption is important, and an OPD with smaller NEP would be able to reduce the required light power. Fuentes-Hernandez et al. developed OPDs with noise performance and NEP comparable with commercial silicon PDs [14]. As the area increased from 0.1 to 1 cm^2^, the noise level, and NEP did not increase significantly. Lower responsivity observed with the flexible device than that with the rigid device was primarily due to reflection losses (~ 30%) caused by the bottom semitransparent Ag/MoO_*x*_ electrode. As shown in Fig. 12c, a large area flexible ring-shaped OPD was fabricated for heart rate detection and showed similar PPG signal quality compared to that obtained using the silicon PD, but with a reduced power consumption of the LED. Park et al. further reported a skin-alike stretchable low-noise OPD fabricated by blending elastomer matrix OSC into the active layer [141]. Lee et al. recently developed a flexible reflective organic pulse oximeter with ultralow power consumption [140], where the OPD is wrapped around each small circular OLEDs (Fig. 12d). Such geometries realized operation power of only a few tens of µW.

### Optical imagers

Optical imagers are required for many applications including cameras, biometric authentication, machine vision, and medical inspection (X-ray). Compared to traditional silicon imaging techniques, OPDs have shown considerable competitiveness in these applications due to their flexibility, easy integration, and low cost [142].

As described in Sect. 2, responsivity, sensing speed and dynamic range are the crucial parameters for evaluating OPDs used for image sensors. Here we briefly introduce the evaluation parameters of an image sensor itself. Firstly, resolution, one of the most important parameters, representing how much detail the imager can capture. Higher resolution implies larger pixel density, which is generally expressed in pixel per inch (ppi). Therefore, the miniaturization of optical sensor and switching element in a single pixel is the precondition for high-quality imaging. However, actual images may be blurred by a high ppi imager, due to crosstalk between individual pixels. Consequently, spatial resolution that defines the discernible two adjacent structures as being distinct from one another (expressed in line pairs mm^−1^), is used to evaluate the contrast and resolution simultaneously. In addition, the readout speed is another important parameter of an imager, depending on the response speed of the optical sensor unit, the switch elements, and the analog–digital conversion circuits. When capturing static information, readout speed is insignificant. However, high reading speed becomes important for dynamic capture. For example, a capture rate of at least 30 frame per seconds (fps) is necessary to record a smooth video for the human eyes. As the resolution increases, the total switching time and reading time also increase accordingly. Therefore, realizing high resolution and high speed simultaneously in imagers is very challenging.

**Fig. 13:**
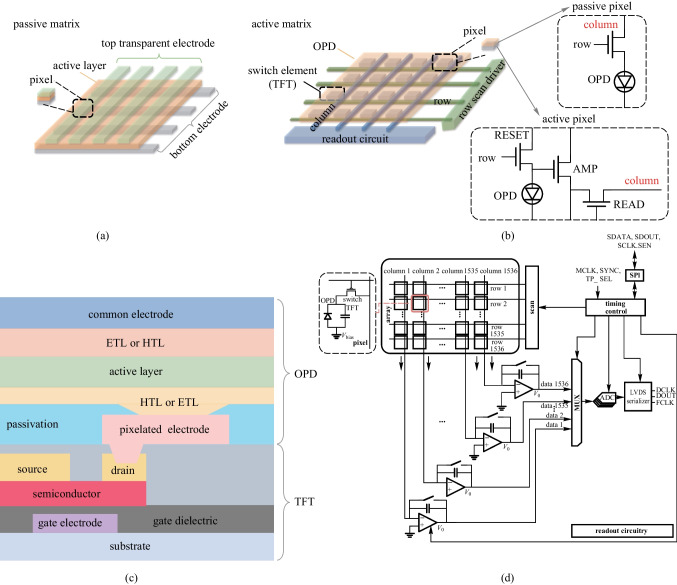
**a** Schematic configuration of passive matrix. **b** Schematic configuration of active-matrix including passive and active pixels. **c** Schematic of the cross-section of the OPD-TFT integration structure. **d** Schematic of an organic active-matrix imager array and the readout circuitry. Reproduced with permission from Ref. [143]

As shown in Fig. 13a, a simple OPD imager array can be built by sandwiching the active layer between orthogonal row bottom electrodes and column top electrodes, which is a so-called passive matrix [144]. However, as the leakage currents of unaddressed pixels would influence the output signal in this scheme, it is hard for a passive matrix to realize high-resolution and high-quality imaging. Therefore, an active-matrix imager array is typically used for high-resolution and large-area imaging applications (Fig. 13b). In active-matrix imagers, the switch elements and OPD are vertically stacked, and TFTs are commonly used as switch, although blocking diodes and complementary metal oxide semiconductor (CMOS) have also been proposed. Taking the integration of OPD and TFT as an example, the top electrodes of all OPDs are connected to a common electrode, while the bottom electrode are pixelated and connected to a common readout line via a TFT switch (Fig. 13c). As shown in Fig. 13d, the readout circuits (ROIC) are connected to the input of a specified readout amplifier. The readout of the flat-panel imager is realized by a line-by-line scan, which is similar to that of active-matrix displays [145]. Depending on the read-out methods, the imager pixel can be classified into two categories: passive pixel sensor and active pixel sensor. The former consists of only a switching element with an optical sensor in each cell and is beneficial for simplifying the production process. However, it is easily affected by wiring noise, which causes low SNR and crosstalk. As a result, the pixel density of this type of imager sensor usually has to be sacrificed due to the deteriorative SNR with decreasing pixel size. An active pixel sensor is realized by introducing an additional amplifier circuit to the passive pixel sensor, which can directly amplify the sensor signal. Figure 13b shows the most typical 3 T-active pixel sensor, containing a reset switch, a select switch, and an amplification circuit. Consequently, SNR deterioration can be mitigated. However, most reported organic imager arrays have employed active-matrix based on the passive pixel sensors, owing to the challenges in fabricating high-resolution complex circuits in active pixel sensors.

**Fig. 14:**
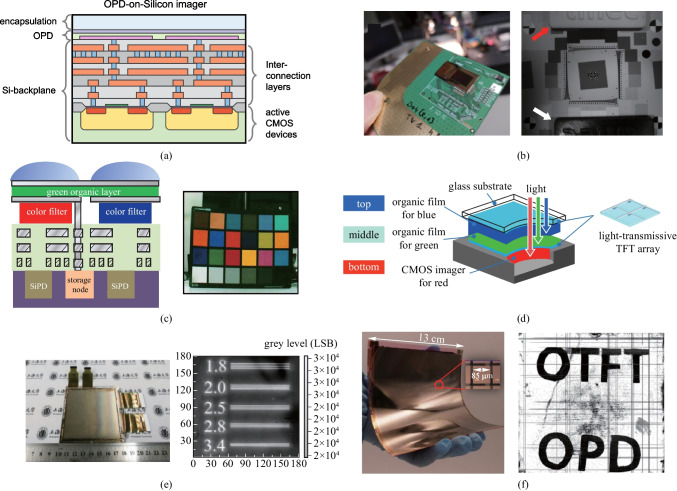
**a** Schematics of the cross-section of OPD-silicon imager. **b** NIR-OPD imager using CMOS as readout circuit. This imager could distinguish two objects made from different materials under NIR illumination. Reproduced with permission from Ref. [146]. **c** Simplified schematics of the cross-section of the hybrid stacked image sensor: a green OPD layer is located on top of the blue and red color filters, through which bottom silicon PDs selectively detect blue and red light. A full-color image was taken by this hybrid camera. Reproduced with permission from Ref. [147]. **d** Schematics of a three-layer-stacked image sensor: a top blue OPD, a middle green OPD, and a bottom red layer of CMOS imager. TFT arrays were used to read out photoelectrically converted signals detected by the OPDs. Reproduced with permission from Ref. [148]. **e** Active-matrix imager fabricated by integrating OPDs onto α-Si TFTs could realize high spatial resolution of over 3.4 line pairs/mm. Reproduced with permission from Ref. [149]. **f** All organic integration for large-area and flexible high-resolution imaging: 1536 × 1536 pixels in 130 mm × 130 mm active imaging area. Reproduced with permission from Ref. [143]

**Fig. 15:**
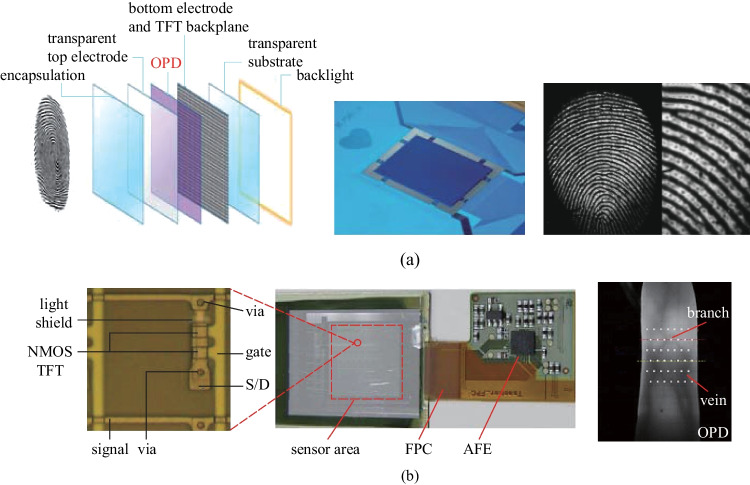
Organic imagers for biometric authentication. **a** Reflective fingerprint scanner consisting of a backlight, a TFT backplane, and an OPD front plane realized a high resolution of 508-ppi. Close-up image of the fingerprint after offset/gain correction and interpolation of nonfunctional pixels, which enabled resolving level 3 details in biometric parameters. Reproduced with permission from Ref. [161]. **b** Organic conformable imager comprising LTPS TFTs and NIR OPDs for vein recognition under NIR illumination. Reproduced with permission from Ref. [31]

CMOS-based Si photodetectors dominate in the camera market share owing to their high performance, technical maturity, and high- level integration with electronics. As shown in Fig. 14a, unlike conventional Si image sensors, organic image sensors separate the photosensitive part (OPD) from the signal processing part (Si-backplane) [150, 151]. Due to the advantage of CMOS, the integration of OPD and CMOS is feasible for developing high-resolution imagers. With the increasing demand for multispectral information acquisition, NIR multispectral imaging technology has received extensive attention [152–155]. Yang et al. developed a NIR-OPD with low dark current by interface engineering and monolithically integrated it with a silicon CMOS readout circuit to build an image sensor [146]. The image sensor offers a focal plane array consisting of 512 × 768 pixels with a 5 μm pixel pitch to achieve high-definition imaging. Figure 15(b) shows the captured picture under NIR illumination, in which the two objects (indicated by red and white arrows) made from different materials have black and white contrast that cannot be distinguished by a standard visible-light camera. Besides NIR imaging, color-selective OPDs are promising in full-color imaging applications [156, 157]. Many leading companies have focused on the development of new-concept cameras because of the immense commercial application potentials [158]. For example, Samsung reported a novel image sensor architecture realized by superposing green-selective OPD onto CMOS circuits and Si photodetectors covered by blue and red color filters (Fig. 14c) [147, 159]. This stacked structure enhanced sensitivity and resolution compared to traditional COMS cameras with a standard Bayer filter array. A full-color image was obtained by this hybrid integrated camera consisting of 5 megapixels. Sakai et al. reported another three-layer-stacked full-color camera comprising two OPDs with TFTs and a CMOS image sensor that is compatible with a small pixel size of 20 μm (Fig. 14d) [148]. The camera has the advantage of no color filter array, and can record videos at 60 fps with 320 × 240 pixels. Low spectral crosstalk and linear output characteristics of the camera result in superior color reproducibility.

The large area scalable manufacturing of OPDs is important for imager applications. By taking the advantage of TFT backplane technology, OPD with high sensitivity or customized response wavelength can be fabricated on various TFT backplanes for large-area imagers [160]. Li et al. integrated OPD onto α-Si TFT backplane to fabricate a large-area, flat-panel image array [149]. The number of pixels and active area were 510 × 470 and 75.0 mm × 81.0 mm, respectively. A newly synthesized p-type CuCrO_2_ nanomaterial was used as HTL to reduce dark current (6.48 × 10^–8^ A/cm^2^), which enhances imaging capability at low light intensity. As shown in Fig. 14e, the imager showed excellent spatial resolution and could distinguish the line pair phantom of 3.4 line pairs/mm. In addition to the large-area producibility, the mechanical flexibility is also attractive for organic imagers. Hou et al. fabricated a flexible large-area active-matrix imager by integrating OPD on the OTFT backplane through a facile blade-coating process. The highest process temperature was controlled to be less than 85 ℃ [143]. As shown in Fig. 14f, the imaging array had the largest size of 130 mm × 130 mm and the maximum number of pixels of 1536 × 1536 in most reported works.

Fingerprint authentication is one of the most widely used biometric technologies. Typically, a resolution of 200 ppi is high enough to unlock mobile phones by fingerprint authentication. However, a higher resolution is preferable for more accurate imaging and thus a greater margin of safety. Tordera et al. integrated OPD on a 508-ppi IGZO dual-gate self-aligned TFT backplane using a slot-die-coating process (Fig. 15a) [161]. The OPD devices showed high D* exceeding 10^12^ Jones, with a very low dark current of about 10^−7^ mA/cm^2^ at − 2 V, and a high-quality optical fingerprint scanner capable of imaging in reflection mode was realized. The fingerprint raw image after offset or gain correction and interpolation of nonfunctional pixels could resolve level 3 details in biometric parameters, where the sweat pores were clearly visible. A cross-section through the center of an image showed good contrast of about 7000 least significant bits (LSB). The implementation of personal identity verification in display terminals is burgeoning in the booming mobile internet. Currently, small and rigid fingerprint imagers hinder the application of imaging in the “full display”. Kamada et al. incorporated an OPD in the same pixel together with OLEDs through side-by-side patterning [162]. Although the resolution of the display was decreased, fingerprint identification or other functionalities may be provided, paving the way to interactive display. OPDs-based imagers have been commercially tested in recent years. The Isorg and FlexEnable companies are continually working toward the commercialization of all-organic imagers, which combines the flexible OTFT array with OPD. A “fingerprint-on display” solution has been proposed by Isorg company for full smartphone display allowing multiple fingers authentications. The fingerprint module is thinner than 300 µm with a matching time of less than 200 ms and a false acceptance ratio of less than 1/50000.

Besides fingerprint authentication, vein recognition is an emerging approach for biometric authentication, and relies on the high resolution and high uniformity of the image detected by NIR light illumination. As a wearable imager, flexibility is also required to be conformal to the skin. Normally, health monitoring needs to detect weak biological signals at a fast speed, while personal identity verification needs to acquire biometric authentication information in a static and clear image. Considering the requirements above, the noise current of the sensor needs to be controlled while the imager should be flexible, feasible to cover human organs, and can acquire high-resolution image at a high speed. As shown in Fig. 15b, Yokota et al. integrated LTPS TFT with NIR sensitive OPD; the fabricated imager has a resolution of 508 ppi, and imaging speed of 41 fps [31]. This made the imager feasibile to monitor the health conditions, obtain biometric information, and measure bio-signals simultaneously. More importantly, the high sensitivity of OPD enabled imager to readout photocurrent of less than 10 pA, which is important in biometric imaging.

**Fig. 16:**
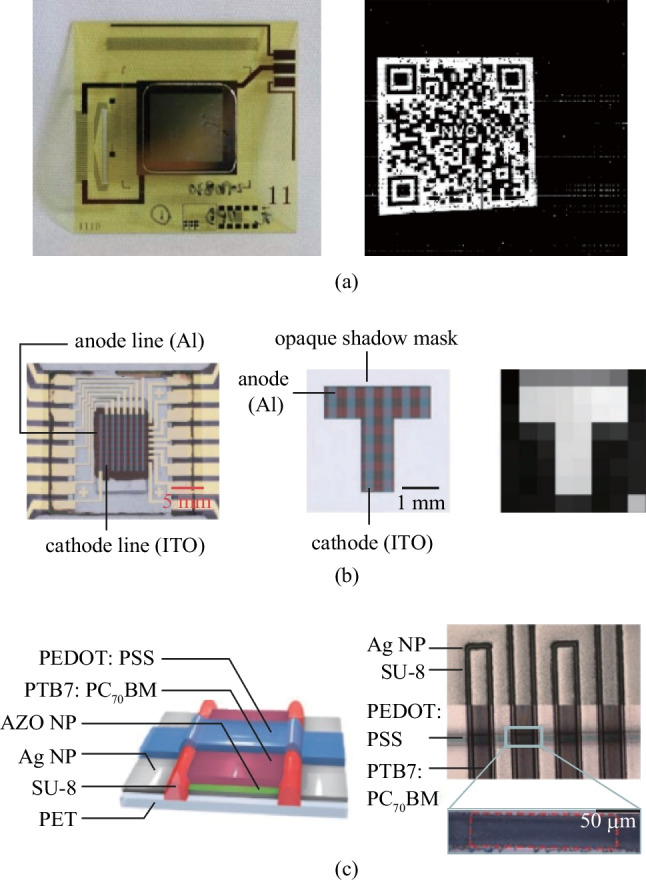
Organic imagers for machine vision. **a** Small-area rigid organic active-matrix imager for QR code recognition. Reproduced with permission from Ref. [163]. **b** Passive-matrix imager stacking a rectifying unit on top of the photomultiplication OPDs enabled imaging under weak light illumination. The 8 × 8 imager array could realize letter recognition when a T-shaped shadow mask is applied. Reproduced with permission from Ref. [144]. **c** Fully digitally printed organic passive-matrix imager. Reproduced with permission from Ref. [164]

Organic imagers have facile integration processes and excellent mechanical flexibility, and are promising in machine vision for distributed and real-time pattern recognition. For example, Xu et al. took the advantage of small leakage current of oxide TFT, and integrated an OPD on indium zinc oxide TFT with the total electronic noise less than 683 e^−^ [163]. As shown in Fig. 16a, the imager had 256 × 256 pixels and a resolution of 500 ppi, and successfully acquired a clear quick response (QR) code, which has been widely used in mobile payment and URL access. Although low resolution of passive matrix limits their application in high-definition imaging, low cost makes it appropriate for simple pattern recognition. As the output signal of the addressed pixel is affected by the leakage currents from all pixels at the same readout line, high SNR is essential here. Wu et al. reported a simple passive matrix imager without an amplifier [144]. A highly responsive organic image sensor based on monolithic, vertically stacked two-terminal pixels was introduced. The OPD showed high responsivity (> 40 A/W), and low dark current (10^−7^ A/cm^2^) at the same time. As shown in Fig. 16b, the 8 × 8 imager array uses a rectifying unit of PEDOT:PSS/P3HT/NaF/Al on top of the photomultiplication OPDs unit to improve the rectification. The disadvantage of this imager array is the high operating voltage of up to 20 V, which is an intrinsic issue with photomultiplication OPDs. A clear image of a “T” shaped pattern was obtained even under a low light intensity of 1 µW/cm^2^, because of the minimized crosstalk. In addition, a fully digitally printed organic passive matrix imager was successfully demonstrated by Eckstein et al. (Fig. 16c). However, the difficulties in production technology and unstable electrodes limited its practical applications [164].

**Fig. 17:**
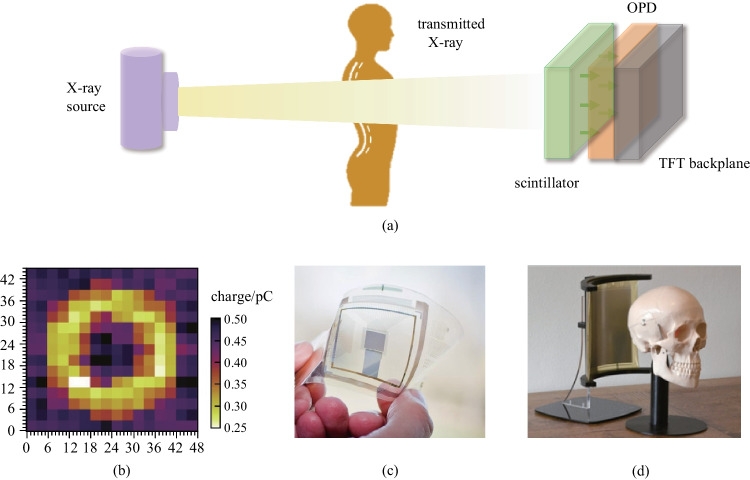
Internal information acquisition by X-ray imaging. **a** Illustrated working principle of X-ray imaging. **b** Passive-matrix imager for X-ray imaging of a standard metal washer under a 50 keV X-ray beam. Reproduced with permission from Ref. [165]. **c** Low-weight, flexible, and thin active-matrix imager for X-ray imaging. Reproduced with permission from Ref. [34]. **d** Prototype of curved organic imager approaching cone beam computed tomography X-ray imaging. Reproduced with permission from Ref. [166]

Another important biological imaging function is getting inside-body information using X-rays. The transmittance of X-rays differs in due to tissue type (muscles, organs, and bones) and tissue thickness. Therefore, inside-body information can be transferred into a contrast image by detecting the ratio of the X-ray transmission. Organic X-ray imagers adopt an indirect conversion process because of the extremely low absorption coefficient of OSCs. Figure 17a shows the structure for realizing indirect conversion, in which a scintillator that absorbs X-rays and emits visible light is placed on top of the organic imager. With such structures, objects can be successfully imaged under X-ray illumination.

Posar et al. demonstrated a prototype of an OPD array coupled with a plastic scintillator for X-ray imaging [165]. The X-ray response is energy independent between 50 keV and 1.2 MeV, and the device showed a fast temporal response and an equivalent detection sensitivity to its inorganic counterpart**.** Multiple 2 mm × 2 mm OPD pixels based on P3HT and o-IDTBR were used to build the passive matrix imager. The 2D X-ray image a flat aluminum annulus-shaped washer shows high contrast (Fig. 17b). Imaging on curved surfaces and 3D imaging are difficult to achieve with commercial X-ray detectors based on rigid silicon PDs. Gelinck et al. fabricated an active-matrix X-ray imager using OPDs and OTFTs [34]. The low-weight, flexible, and shatterproof X-ray detectors of ca. 400 μm thickness were achieved (Fig. 17c). X-ray images could be recorded at dose levels that arenormally used in medical applications (0.27 mGy/s). As shown in Fig. 17d, a polyimide foil-based curved digital X-ray image sensor was developed by van Breemen et al. for cone beam computed tomography X-ray imaging [166]. The flexible image sensor with a 32 cm curvature radius had 480 × 640 pixels in a large area of 6 cm × 8 cm. An exquisite 3D image of a piece of bone was successfully reconstructed by using the X-ray image sensor, demonstrating the promising future of OPD-based X-ray detectors in medical applications.

### Spectrometers

Optical spectrometry is a powerful analysis tool in scientific research and in industry. Traditional laboratory spectrometer systems with strong analytic ability have bulky optical components, moving parts, and long path lengths, which cannot meet the requirement of the rapidly growing applications of mobile and portable detection [167]. Miniaturized spectrometers are very attractive in the applications where low cost, lightweight, and tailorable optoelectronic characteristics are preferred. Several strategies have been discussed by Yang et al. [168]. One approach is using an array of PDs, where each PD is sensitive at a specific wavelength, i.e., possesses narrow band response characteristics, and is continuously tunable for detection of different wavelengths. OPDs are promising because of their properties of narrowband detection without color filters, low-cost fabrication, and ease of integration. The working principle of miniaturized spectrometers is as follows. Firstly, the incident light from a certain light source is either being reflected or absorbed by the sample. Then the light waves reflected by or transmitted through the sample are detected by PDs, and the collected data is analyzed to identify the ingredients according to the characteristic absorption spectrum of materials. For example, many characteristic absorption features (in the NIR region) of organic compounds can be used in both qualitative and quantitative analysis. Such identification allows for a wide application in daily life and industry, especially for on-site automatic quick testing.

**Fig. 18:**
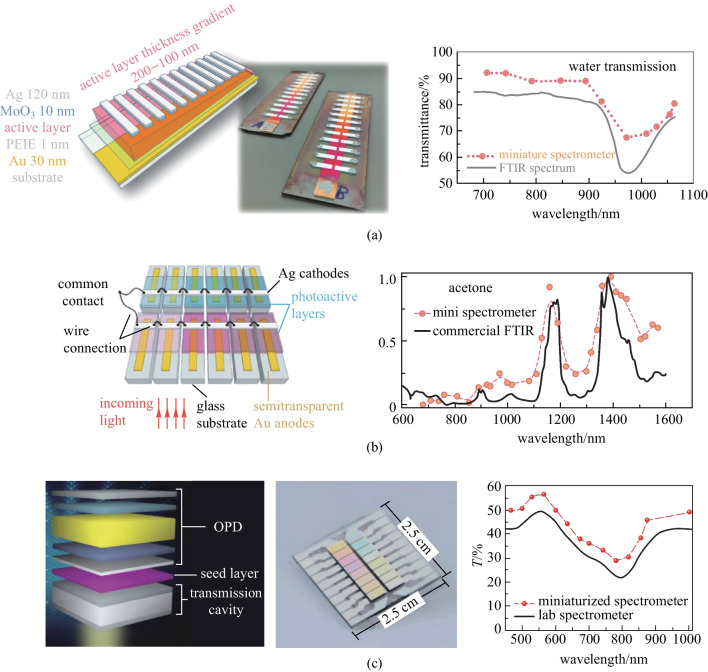
OPD-based miniaturized spectrometer. **a** Altering the thickness of the active layer by blade-coating to fabricate a resonant cavity-enhanced OPDs array. The on-chip miniaturized NIR spectrometer and a commercial Fourier-transform infrared spectrometer were used to measure the water transmittance spectrum, and similar results were obtained. Reproduced with permission from Ref. [36]. **b** Combining a series of cavity-enhanced OPDs with continuously tuned detection wavelengths to build a miniaturized spectrometer. The miniaturized spectrometer with thirty-one channels was used to measure the absorption spectra of acetone. The obtained results were similar to that obtained with a commercial Fourier-transform infrared spectrometer. Reproduced with permission from Ref. [104]. **c** Applying “external” resonant cavity to tune narrowband detection. The miniaturized spectrometer with a dimension of 2.5 cm × 2.5 cm containing sixteen separated channels was used to measure the transmission spectrum of a semi-transparent organic solar cell. Similar results was obtained compared with that obtained with a professional laboratory spectrometer. Reproduced with permission from Ref. [35]

Up to now, successful demonstration of OPD in miniaturized spectrometers applications relies on the coupling of resonance optical cavity. The most representative work was reported by Tang et al. in 2017 [36]. As shown in Fig. 18a, a resonance cavity consisting of Au and Ag metal electrodes as mirrors, and active layer as spacer were employed to fabricate tunable wavelength-selection OPDs. The required narrow spectral resonances were achieved by exploiting the weak sub-gap absorption of intermolecular charge-transfer (CT) state of donor and acceptor. To enhance the absorption strength of the CT state, which is proportional to the area of donor/acceptor interface, a semi-crystalline polymer, PBTTT, was applied to gain a highly intermixed donor/acceptor BHJ as desired. Remarkable EQE values of 20%–40% with FWHM values as small as 20 nm at 700–1000 nm were obtained by this approach, which is comparable with commercial inorganic counterparts. Finally, a prototype miniaturized spectrometer with gradient active layer thickness was fabricated by blade-coating. The spectrometer with sufficient spectral resolution successfully measured the transmittance spectrum of water, indicating innovative application areas such as moisture detection.

Recently, Yang et al. also utilized a similar cavity concept to build an OPDs module with continuously tuned detection wavelengths over a wide wavelength range from 660 to 1510 nm [104]. The cavity thickness was adjusted by varying the thickness of ZnO spacer (served as ETL here). Considering the tradeoff between the spectral selectivity and responsivity originating from the microcavity mechanism, they found that satisfactory device performance could be expected only if the absorption coefficient of the active layer is in the range between 10^−3^ and 10^−5^ nm^−1^ by using transfer matrix simulations. As result, a low-bandgap polymer PCDTPTSe was developed to meet requirement of absorption coefficient. The FWHM values of the detection peak could be controlled between 20 and 40 nm in a broad spectra range. As illustrated in Fig. 18b, a proof-of-concept miniature NIR spectrometer was eventually constructed by combining a series of cavity-enhanced OPDs. The resolution was sufficiently high for resolving the absorption features of water, ethanol, and acetone.

The spectrum selectivity were realized by using an “internal” resonant cavity in the above two cases, with balance of the optical and electronic properties. Xing et al. reported a novel concept by integrating an innovative transmission cavity structure with OPDs at the light incident side, as shown in Fig. 18c [35]. The use of an “external” resonant cavity can reflect untargeted incoming photons without changing the optimized optoelectronic structure of the OPDs. The continuously tunable narrowband OPDs were achieved by simple variation of the spacer layer thickness in the transmission cavity and appropriate donor–acceptor combinations. Outstanding spectrum selectivity (FWMH of approximately 40 nm) in the wavelength range of 400–1100 nm and ultrahigh D* above 10^14^ Jones (calculated by thermal noise) were achieved by using this design. As shown in Fig. 18c, a prototype miniaturized spectrometer consisting of numerous such cavity-enhanced OPDs was successfully fabricated on a single substrate by a full vacuum processes. The output signals is comparable to that of a commercial laboratory spectrometer when measuring a semi-transparent organic solar cell.

Since miniaturized spectrometers are so attractive for mobile applications, narrowband OPDs are already on the way to commercialization, in particular for mobile spectroscopy. The handheld spectrometer produced by Senorics GmbH integrates sensors (area of 11 mm × 11 mm), NIR light sources, and electronics, and can provide sixteen wavelength channels with equal interval from 1200 to 1700 nm. The solid or powder samples can be easily analyzed by contacting samples to the measurement window (diameter of 5 mm), followed by data transmission via Bluetooth.

### Light communications

Nowadays, with the development of mobile Internet and the standardization and industrialization of 5G technology, communication technology is constantly being renovated to realize virtual reality, smart city, cloud computing, autonomous driving, and so on. New challenges of the mass data transmission in communication networks have arisen with the explosive growth of big data services [169, 170]. Because of the ever-dwindling wireless communication spectrum resources, and emerging demand for wireless communication, visible light communication (VLC) with visible light as the carrier of information, is picking up focus as an alternative communication technology [125, 171]. The ultra-large bandwidth, measured in terahertz (THz), means that VLC has potential for high-speed communication, and transmission rates of over Gigabits per second (Gbps) have been demonstrated. The ability to offer data communications and solid-state lighting simultaneously makes VLC more promising in applications [21].

**Fig. 19:**
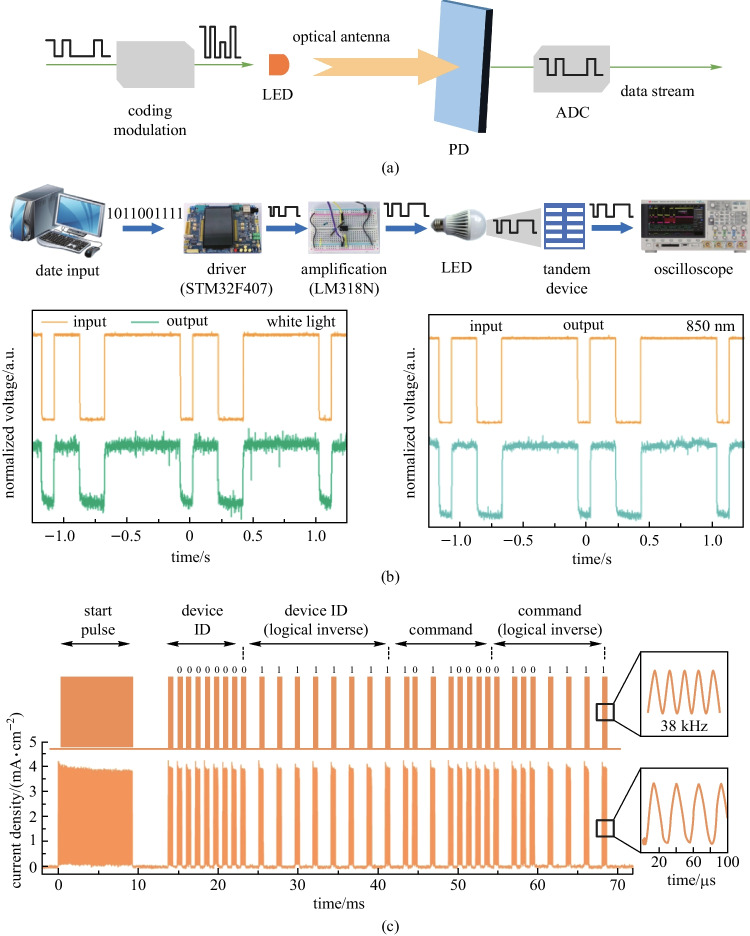
OPD-based light communication systems. **a** Working principle of a basic architecture of point-to-point light communication system. **b** Tandem OPD could convert the input light signals covering from visible to NIR precisely into an electrical signal, which is displayed on an oscilloscope. Reproduced with permission from Ref. [126]. **c** NIR communication: NIR-OPD could transcribe the message accurately from a commercial NIR remote control. Reproduced with permission from Ref. [41]

The two main components of VLC systems are transmitters and receivers, and the working principle of VLC can be simplified as the process shown in Fig. 19a [172, 173]. Firstly, LEDs are commonly used as transmitters in most research because of their easy coupling, security, and low cost. The driving current carrying encoded information can be generated by a control chip integrated with the LED, and manipulates the intensity of the output light. On account of the high operating frequency of LED the light intensity variation is imperceptible to human eyes. At the receiver side, PDs are responsible for capturing the light signal and converting it into electrical current. Finally, the data flow is recovered by an analog–digital converter (ADC). A higher response speed with the larger response bandwidth of PDs is required for light communications than that for other applications. PDs based on inorganic semiconductors are mostly used in receivers in VLC, but the requirement of cut-off filters to prevent NIR light and complex fabrication processes increase the cost of devices. OSC can be easily designed with matched absorption spectrum with indoor white light LEDs, so OPDs are a promising candidate to meet the requirement of the increasing demand for applications with high flexibility and wavelength selectivity. The relatively narrow response bandwidths of OPDs are still challenges, due to the low charge carrier mobility of OSCs compared to Si. Therefore, OPDs are more attractive in the application scenarios where high communication speed is insignificant and low power consumption is more important, such as for the Internet of Things (IoT) [174, 175].

The possibility of OPDs as receivers in VLC systems came up as early as a decade ago, and was first demonstrated by Ghassemlooy et al. in 2013 [176]. The active layer of OPD was deposited by spray coating a blend of P3HT as the donor material and PC_61_BM as the acceptor material. Four individual devices with a 1 cm^2^ active area were fabricated on a rigid glass substrate. The response bandwidth of OPDs increased from 56 to 160 kHz when varying light intensity from 10 to 300 μW/cm^2^ because of the relative small amounts of charge carriers to interface traps at low light intensity. With an artificial neural network (ANN)-based equalization, the first megabit per second (Mbits/s) VLC link using a white phosphor LED as the transmitter and the OPD as receiver was experimentally demonstrated, which showed the potential of OPDs for large-area optic-free VLC systems.

Liu et al. reported a fast-response tandem OPD with a wide detection range from 300 to 1000 nm to demonstrate light communication [126]. Figure 19b shows the optical communication process. The programmable driver converted the digital data to voltage square wave, which was amplified to drive various LEDs. The optical signal was received by the tandem OPD and converted into electrical signal, which is displayed on an oscilloscope. Due to the excellent performance in responsivity and response speed, the original input waveform of light signals was accurately restored, demonstrating digital signal communication using visible to NIR light.

NIR communication is also a practical application for OPDs. The NIR responsive OPD developed by Babics et al. could accurately transcribe the message carried by a light signal around 910 nm that is emitted from a commercial remote control [41]. A new NFA, O4TFIC with absorption over 1000 nm was developed to provide responsivity of 0.50 A/W at 890 nm without bias. Since the NIR communication protocol defines the frequency range of between 38 and 50 kHz for remote control, the decay of the device response for different frequencies of a sinusoidal NIR signal was investigated. PM6:O4TFIC combination demonstrated minor damping up to 10 kHz, and the damping were less than 1 dB at the NIR communication frequency range, which means about 90% output signal amplitude compared to that under continuous wave illumination. As shown in Fig. 19c, an NEC transmission protocol consisting of several basic parts with a total duration of 67.5 ms was used to demonstrate the potential of OPDs for NIR communication. The tested OPD exhibited a high ratio of on/off, thus the signal sequence with a clear distinction between logical “0” and “1” can be captured by an oscilloscope [41].

**Fig. 20:**
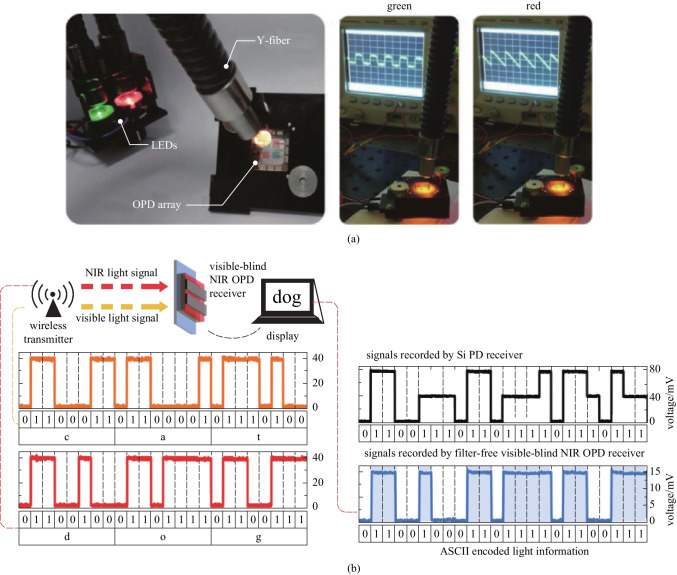
Wavelength-selective OPDs for encrypted optical communication and anti-interference light communications. **a** Encryption communication by OPDs array consisting of different wavelength-selective units: Different signals were successfully read out without additional optical filters from the multiplexed two-color lights via an optical Y-Fiber. Reproduced with permission from Ref. [40]. **b** Decode information in dual-channel light communications: The light information of the word “dog” (in NIR light) encoded in ASCII was decoded by the visible-blind NIR OPDs. Reproduced with permission from Ref. [105]

Realizing band-selective photodetection without using optical filters is one of the advantages of OPDs, thus making OPDs promising candidates in encryption communication. Recently, Strobel et al. developed inkjet-printed color-selective OPDs, and successfully demonstrated demultiplexing of optical signals simultaneously transmitted at different wavelengths by a 2 × 2 OPD array [40]. The OPDs exhibited up to MHz response bandwidth and had complementary responsivity in the green and red spectral range. Figure 20a illustrates the demonstration of a multichannel VLC system. Two LEDs, green- and red-emitting, were driven independently by different alternating current (AC) signals and used as transmitters. The multiplexed signals transmitted through an optical Y-fiber were demultiplexed by the printed OPDs array based on selective detection. Finally, square and triangular signals driving LEDs of different color were successfully read out without additional optical filters. This simple approach offers a general method for realizing encrypted optical communication by printed electronics in the future.

The wavelength-selective property also makes OPDs free of visible light interference in light communications. For example, Lan et al. presented a new approach to fabricating the filter-free visible-blind NIR OPDs for the application of decoding the NIR signal in dual-channel light communications [105]. The target device had high SRR and response bandwidth of approximately 100 kHz, which is suitable for efficient demultiplexing in light communications. The light signals containing both the word “cat” (visible light) and the word “dog” (NIR light) encoded in ASCII were broadcast simultaneously by the dual-channel wireless transmitter. The NIR light carrying the encoded signal was successfully decoded by the OPD receiver, as shown in Fig. 20b, but was failed when a broadband a silicon PD was used. Furthermore, customizable light communications systems can be realized by using different combinations of depletion layers and BHJ layers in OPDs. This work implies that OPDs are promising in indoor IoT applications, where visible light should not interfere with NIR light communications.

## Conclusion and outlook

Device designs and technologies for improving the key performance metrics of OPDs for various applications, including wearable medical diagnostics, optical imagers, spectrometers, and light communications, are reviewed in this work. The demonstrated prototype systems prove the technical advantages of OPDs. However, several challenges remain for practical OPD applications.

Firstly, although many high-performance photo-sensitive organic semiconductors have been developed, effective processes are not yet developed for incorporating these materials and their matched interfacial layers into device stacks for large area scalable manufacturing. OPDs can be fabricated through vacuum thermal evaporation or solution coating/printing. The former method can leverage the established material and equipment base of OLED manufacturing in the display industry, but has issues of high cost and limited scalability. The latter is more popularly used, but needs to carefully select the material stack and proper coating or printing methods for layer-to-layer integration and accurate control the film thickness and morphology during fast processing over a large area.

Secondly, the material stacks used for OPDs are mostly derived from the extensive OPV research, but the operation mode of OPDs is quite different from that of OPVs and is more susceptible to dark current. Therefore, the performance of OPDs based on the same materials might be more sensitive to ambient influence. It is important to understand the instability mechanisms of OPDs and develop technologies to achieve air-stable OPDs.

Thirdly, since the required performance metrics vary for different applications, it is important to establish application-driven characterization standards for OPD materials and devices. With such standards, the community can follow the clear directions for optimal co-design of materials and devices to meet the application requirements, and the performance of each OPD can also be fairly evaluated.

Last but not the least, the merits of low-temperature processing and excellent intrinsic flexibility would enable OPDs to be used for developing free form photo-sensing devices and systems on a wide range of substrates. For that, however, both the mechanical structure design and the processing techniques need to be developed. Reliably integration of the devices with external electronic systems is also a challenge.
