# Correction to: Organic photodiodes: device engineering and applications

**DOI:** 10.1007/s12200-022-00057-w

**Published:** 2022-12-30

**Authors:** Tong Shan, Xiao Hou, Xiaokuan Yin, Xiaojun Guo

**Affiliations:** School of Electronic Information and Electrical Engineering, Shanghai Jiao Tong University, Shanghai, 200240 China

**Correction to: Frontiers of Optoelectronics (2022) 15:49** **https://doi.org/10.1007/s12200-022-00049-w**

Following publication of the original article [1], the authors identified an error in the author name of Tong Shan. The given name and family name were erroneously transposed.

The incorrect author name is: Shan Tong.

The correct author name is: Tong Shan.

The author group has been updated above and the original article [1] has been corrected.
